# Identification of Multifunctional Putative Bioactive Peptides in the Insect Model Red Palm Weevil (*Rhynchophorus ferrugineus*)

**DOI:** 10.3390/biom14101332

**Published:** 2024-10-19

**Authors:** Carmen Scieuzo, Roberta Rinaldi, Fabiana Giglio, Rosanna Salvia, Mohammed Ali AlSaleh, Jernej Jakše, Arnab Pain, Binu Antony, Patrizia Falabella

**Affiliations:** 1 Department of Basic and Applied Sciences, University of Basilicata, Via dell’Ateneo Lucano 10, 85100 Potenza, Italy; carmen.scieuzo@unibas.it (C.S.); roberta.rinaldi@unibas.it (R.R.); fabiana.giglio@unibas.it (F.G.); r.salvia@unibas.it (R.S.); 2 Spinoff XFlies s.r.l, University of Basilicata, Via dell’Ateneo Lucano 10, 85100 Potenza, Italy; 3 King Saud University, Chair of Date Palm Research, Center for Chemical Ecology and Functional Genomics, College of Food and Agricultural Sciences, Riyadh 11451, Saudi Arabia; malsaleh@ksu.edu.sa; 4 University of Ljubljana, Biotechnical Faculty, Agronomy Department, SI-1000 Ljubljana, Slovenia; jernej.jakse@bf.uni-lj.si; 5 King Abdullah University of Science and Technology (KAUST), Bioscience Programme, BESE Division, Thuwal, Jeddah 23955-6900, Saudi Arabia; arnab.pain@kaust.edu.sa

**Keywords:** transcriptome, antimicrobial peptides, ACPs, AFPs, AVPs, bioinformatic tools, gene duplication

## Abstract

Innate immunity, the body’s initial defense against bacteria, fungi, and viruses, heavily depends on antimicrobial peptides (AMPs), which are small molecules produced by all living organisms. Insects, with their vast biodiversity, are one of the most abundant and innovative sources of AMPs. In this study, AMPs from the red palm weevil (RPW) *Rhynchophorus ferrugineus* (Coleoptera: Curculionidae), a known invasive pest of palm species, were examined. The AMPs were identified in the transcriptomes from different body parts of male and female adults, under different experimental conditions, including specimens collected from the field and those reared in the laboratory. The RPW transcriptomes were examined to predict antimicrobial activity, and all sequences putatively encoding AMPs were analyzed using several machine learning algorithms available in the CAMP_R3_ database. Additionally, anticancer, antiviral, and antifungal activity of the peptides were predicted using iACP, AVPpred, and Antifp server tools, respectively. Physicochemical parameters were assessed using the Antimicrobial Peptide Database Calculator and Predictor. From these analyses, 198 putatively active peptides were identified, which can be tested in future studies to validate the *in silico* predictions. Genome-wide analysis revealed that several AMPs have predominantly emerged through gene duplication. Noticeably, we detect a newly originated defensin allele from an ancestral defensin via the deletion of two amino acids following gene duplication in RPW, which may confer an enhanced resilience to microbial infection. Our study shed light on AMP gene families and shows that high duplication and deletion rates are essential to achieve a diversity of antimicrobial mechanisms; hence, we propose the RPW AMPs as a model for exploring gene duplication and functional variations against microbial infection.

## 1. Introduction

Antimicrobial peptides (AMPs) are small bioactive proteins naturally produced by all living organisms. They are essential components of the innate immune system, serving as the first line of defense against microbial infections in eukaryotes. In prokaryotes, AMPs are produced as a competitive strategy to inhibit the growth of other microorganisms [1,2,3].

The growing interest in AMPs as potential new drugs stems from pressing social and health emergencies, including rising antibiotic resistance, resistance to antineoplastic chemotherapy, and the emergence of infectious diseases, particularly zoonoses of viral and bacterial origin [4,5]. By 2050, indeed, antibiotic resistance could lead to the death of one person every three seconds, overcoming cancer as a leading cause of mortality [1,6]. The study and the exploration of alternative approaches to antibiotic use will greatly enhance quality of life, significantly improving public health and the healthcare system, while also providing substantial economic benefits [1].

AMPs are small/medium-sized molecules, generally ranging from 5–50 residues and play crucial roles in defense systems [7]. They have a low molecular weight, and most are cationic, showing amphipathic structural configuration characterized by the presence of both hydrophobic and hydrophilic regions [8]. These peptides primarily exert their antimicrobial effect by disrupting microbial membranes, making it difficult for microbes to develop drug resistance. Several models have been proposed to explain the membranolytic mechanism underlying the antimicrobial effects of AMPs [9]. The barrel-stave model describes how peptides laterally insert and diffuse through the lipid bilayer, arranging into helices and forming barrel-like channels that span the membrane [10]. The toroidal pore model involves peptide molecules rotating and inserting into the membrane bilayer, causing a rapid change in membrane conformation and creating a toroidal-shaped pore [11,12]. Finally, the carpet model describes peptides lying parallel to the membrane surface, forming a “carpet” that disrupts the membrane bilayer structure in a detergent-like manner at certain peptide concentrations, leading to micelle formation [6]. Although all living organisms produce AMPs, insects, with their extraordinary biodiversity, represent an almost inexhaustible source of these molecules [13,14]. They hold great potential to enhance the pharmacopeia, which is increasingly depleted of effective therapeutic agents [15,16].

The regulation and production of AMPs in insects are primarily due to the Toll pathway, which is activated by the extracellular cytokine-like polypeptide Spätzle. This pathway is involved in the detection of antigens such as fungi and Gram-positive bacteria, the induction of cellular immunity, and the release of AMPs by the fat body [17]. Specifically, peptidoglycan recognition proteins (PGRPs) mediate Toll activation, particularly GNBP 1 in the case of a Gram-positive bacterial infection and GNBP 3 when a fungal infection occurs. If Gram-negative bacteria provoke infection, the IMD signaling pathway is activated when specific receptors bind meso-diaminopimelic peptidoglycan-2 (DAP), resulting in a signaling activation cascade that triggers the transcription of specific AMPs [18,19]. Finally, if a viral infection takes place, the Janus kinase/signal transducer and activator of transcription (JAK-STAT) cascade is activated to induce AMP gene transcription [19].

Insect AMPs can be classified according to their structure. The three major structural classes are linear α-helical peptides without cysteine residues, peptides with a β-sheet globular structure stabilized by intramolecular disulfide bridges, and peptides that contain high numbers of specific amino acid residues, such as proline or glycine [13,20,21]. AMPs can be also classified in several structural families like abaecin, apidaecin, apisimin, attacins, bomanin, cecropins, cobatoxin, coleoptericin, defensins, diapausing, diptericins, drosocin, drosomycin, gambicin, gloverin, hymenoptericin, lebocins, metchnikowin, morocin, and ponericins, with new AMPs frequently being discovered [1,9,21,22]. This is easily understood when considering that insects, with around one million known species, represent the class of the animal kingdom with more species than the combined total of all other living organisms. This diversity contributes to their remarkable adaptability to environmental changes and tolerance to a wide range of infections, resulting in an incredible, almost inexhaustible biodiversity in terms of anatomical structures, physiological processes, and behavioral patterns [1,6,7,23].

Defensins are a small family of arginine-rich, cationic peptides. They are mostly stabilized by three disulfide bonds, and their fundamental structural feature is a β-hairpin. They bind directly to the cell membrane and create pore-like membrane defects that allow nutrients and vital ions to escape and are isolated from insect orders such as Diptera, Hymenoptera, Coleoptera, Trichoptera, Hemiptera, and Odonata [24,25]. Cecropins were first isolated from the hemolymph of the giant silk moth *Hyalophora cecropia* (Lepidoptera: Saturniidae, L.), whence the term cecropin derived, and they are mostly isolated from various lepidopteran and dipteran species [26]. The main insect cecropins (A, B, and D), lacking cysteine, can lyse bacterial cellular membranes, inhibit proline uptake, and cause membrane leakage [27]. Attacins are glycine-rich proteins, which were initially discovered in the hemolymph of immunized pupae of *H. cecropia*. Attacins are categorized into two groups based on their amino acid composition: attacins with a basic group and attacins that contain acidic residues. The function of these proteins is to inhibit the synthesis of major outer membrane proteins, thereby compromising the integrity of the cell wall [28]. Lebocins were identified in the hemolymph of the silkworm *Bombyx mori* (Lepidoptera: Bombycidae, L.) after immunization with *Escherichia coli*, and these peptides are proline-rich and O-glycosylated [29]. Diptericins are a family of glycine-rich antibacterial peptides derived from Dipteran hemolymph proteins, primarily targeting the cytoplasmic membrane [30]. Ponericins, peptides extracted from the venom of the predatory ant *Pachycondyla goeldii,* adopt α-helical structures within cell membranes and exhibit hemolytic activity [31].

A comprehensive list of known AMPs from six kingdoms is reported in the Antimicrobial Peptide Database (APD) (http://aps.unmc.edu/AP, accessed on 7 September 2024). They comprise 383 isolated/predicted bacteriocins/peptide antibiotics from bacteria, 5 from archaea, 8 from protists, 29 from fungi, 250 from plants, and 2463 from animals, including genome-predicted and some synthetic peptides (190 predicted and 314 synthetic AMPs) (updated January 2024). The Coleopteran pest in the Curculionoidae family *Rhynchophorus ferrugineus*, also known as the Asian palm weevil or red palm weevil (RPW), is an insect whose larvae destroy almost 40 species of palm trees worldwide [32,33], belonging to 16 genera, and it has been classified as a serious pest on the A2 list according to the EPPO 2024 (https://www.eppo.int/ACTIVITIES/plant_quarantine/A2_list, accessed on 7 September 2024, European and Mediterranean Plant Protection Organization).

Throughout their development, lasting from 25 to 60 days, the larvae and adults of *R. ferrugineus* inhabit an environment composed of fermenting plant fibers, rich in microorganisms. Their survival in such a microbe-rich habitat is closely linked to having a robust and effective immune system [34]. The decision to focus the research on AMPs derived from *R. ferrugineus* is backed by recent studies demonstrating consistent antimicrobial activity of the larvae and adults of this Coleopteran species towards different microorganisms: *Bacillus thuringiensis*, *Bacillus subtilis*, *Enterococcus faecalis*, *Staphylococcus aureus* as Gram-positive bacteria [35,36], and *Escherichia coli* and *Klebsiella pneumoniae* as Gram-negative bacteria [36], as well as two species of nematodes, *Steinernema carpocapsae* and *Heterorhabditis bacteriophora* [32]. Moreover, antifungal activity has been detected in the larvae and adults of Coleoptera towards *Beauveria bassiana* and different species of *Penicillium* [35,36]. Advanced bioinformatic analyses allowed us to first identify putative AMPs in different transcriptomes through functional annotation by comparing them with known sequences deposited in the constantly updated protein databases [2,13]. The identified AMPs were analyzed using the CAMP_R3_ (Collection of Anti-Microbial Peptides) database (https://www.camp.bicnirrh.res.in/, accessed on 7 September 2024) to predict the putative antimicrobial activity, the iACP (a sequence-based tool for identifying anticancer peptides) online tool (https://lin.uestc.edu.cn/server/iACP, accessed on 7 January 2024) to predict the putative anticancer activity, the AVPpred (https://crdd.osdd.net/servers/avppred, accessed on 7 January 2024) server to predict the putative antiviral activity and the Antifp (antifungal peptide prediction) server (https://webs.iiitd.edu.in/raghava/antifp, accessed on 7 January 2024) to predict their putative antifungal activity. Their physicochemical properties were then evaluated with the Compute pI/Mw tool—Expasy (https://web.expasy.org/compute_pi/, accessed on 17 December 2023) and the Antimicrobial Peptide Database Calculator and Predictor (APD3) (https://aps.unmc.edu/prediction, accessed on 7 September 2024) [37,38].

## 2. Materials and Methods

### 2.1. Rearing of Rhynchophorus Ferrugineus and RNA Isolation

The RPWs were collected in 2009 with the direct permission of a cooperating landowner [Al-Kharj region (24.1500° N, 47.3000° E) of Saudi Arabia]. Since then, the laboratory colony has been maintained, as previously described [39,40]. Two weeks after pupation, the cocoons were harvested from the sugarcane stems, individually incubated in round 70 mm × 90 mm plastic jars with perforated screw caps, and checked daily for adult emergence. Ten-day-old unmated adult male and female RPWs were selected (male and female separately). Various body parts (antennae, snout, head, thorax, abdomen, gut, fat body, wings, and legs) were carefully dissected (male and female separately) under a light microscope, after insects were anesthetized using CO_2_ for 1–2 min. Immediately after collection, the tissues were transferred in RNA later solution and then stored at −20 °C until total RNA extraction. The RPW male and female adults from the field were captured alive from the infested and removed date palm tree materials at Al Qassim (25.8275° N, 42.8638° E) in Saudi Arabia, and various body parts were collected by following the method described above.

### 2.2. Total RNA Extraction, cDNA Library, and Illumina Sequencing

For the subsequent RNA extraction, intended for the *de novo* construction of transcriptomes, specimens were collected separately for each sample, comprising different body parts from male and female adults reared in the laboratory, as well as those collected directly in the field. Total RNA was extracted from various tissues (~30 mg) of RPW male and female adults separately using a PureLink RNA Mini Kit (Thermo Fisher, Waltham, MA, USA). A NanoDrop spectrophotometer (Thermo Fisher, Waltham, MA, USA) was used to quantify and check the extracted RNA quality and synthesized cDNA quality. The quantity and quality of the total RNA were validated using a Qubit 2.0 Fluorometer (Invitrogen, Life Technologies, Carlsbad, CA, USA), and RNA integrity was confirmed using a 2100 Bioanalyzer (Agilent Technologies, Santa Clara, CA, USA). After ensuring the quality and the characteristic “hidden break” in the 28S RNA profile using 2100 Bioanalyzer, a paired-end cDNA library preparation using a TruSeq Stranded mRNA preparation Kit (Illumina Inc., San Diego, CA, USA) was prepared, following manufacturer’s protocols, which include the following steps: purification and fragmentation of total RNA, first- and second-strand cDNA synthesis, 3′ end adenylation, adapter ligation, and purification. Finally, the purified and PCR-enriched products were used for cDNA library preparation. The cDNA libraries were validated and quantified using a Qubit 2.0 Fluorometer. The HiSeq Illumina sequencing was performed at the core sequencing facility of the King Abdullah University of Science and Technology (KAUST), Jeddah, Saudi Arabia. Image deconvolution and quality value calculations were performed using Illumina GAPipeline1.3. Illumina adaptors were removed, and low-quality bases were trimmed off with the Trim Reads tool of the CLC Genomics Workbench/Server suite. Filtered paired-end reads were validated through a QC for Sequencing Reads visualization of the same suite. A reference *de novo* transcriptome assembly was constructed with the “*De Novo* Assembly” tool of the CLC Genomics Workbench/Server with default parameters (map reads to contigs function on and using paired read files). The resulting contigs files were functionally annotated using the previously described method [39,40,41,42]. RPW antennae, snout, head, thorax, abdomen, gut, fat body, wings, and legs (field and lab) were uploaded to the National Center for Biotechnology Information (NCBI) under Sequence Read Archive (SRA) and Transcriptome Shotgun Assembly (TSA) accession numbers.

### 2.3. Transcriptomes Assembly and Annotation

The transcriptome assembly and annotation were carried out following the method described in Antony et al. (2024), Gonzalez et al. (2021), and Antony et al. (2016) [39,41,43]. Contigs were annotated based on a local BLAST search using *R. ferrugineus* olfactory protein sequences using Geneious R7 v7.1.9 (http://www.geneious.com, accessed on 7 January 2023). Blast2Go analyses were performed on various tissue transcripts using the BLAST2GO command line tool (*v*1.5) of the CLC. The top blast hit transcript clusters were extracted from the male and female (lab and field) assembled transcriptomes with an in-house command-line script. The reads per kilobase million (RPKM) values [44] and the gene and transcript level quantification were performed, and the transcripts per kilobase per million mapped reads (TPM) value of each gene was calculated manually based on the consensus length of each gene and total read counts. To assess transcriptome completeness, an Arthropoda BUSCO database, consisting of 1066 core genes that are highly conserved single-copy orthologues, was used to query the assembled FASTA files. For this process, the gVolante2 web server (https://gvolante.riken.jp/, accessed on 17 January 2023) was utilized with the following parameters: min_length_of_seq_stats: 1, assembly_type: trans, Program: BUSCO_v2/v3, selected reference_gene_set: Arthropoda.

### 2.4. Multiple Alignment

To generate alignments between amino acid residues of all sequences in each part of the body and identify identical AMPs, the Clustal Omega program (https://www.ebi.ac.uk/jdispatcher/msa/clustalo, accessed on 13 December 2023) was utilized. This tool enables the analysis of sequence patterns conserved through evolution, using the FASTA output format for multiple alignments of protein and nucleic acid sequences [45], and it also allows to detect duplicated sequences. According to physicochemical criteria, residues are color-coded differently to indicate conserved positions in the sequences, as shown in Table 1.

### 2.5. In Silico Analysis for the Antimicrobial, Anticancer, Antiviral and Antifungal Activity Prediction

The Blast2Go software [46] functionally categorized the sequences as antimicrobial peptides, which were translated into the corresponding amino acid sequences via the software Expasy translate tool [47]. Then, the amino acid sequences obtained by the Expasy translate tool (https://web.expasy.org/translate/, accessed on 17 December 2023) were analyzed to detect the possible presence of the signal peptide and pro-peptide, using the software Prop 1.0 (https://services.healthtech.dtu.dk/services/ProP-1.0/, accessed on 17 December 2023) [48] and SignalP 6.0 servers (https://services.healthtech.dtu.dk/services/SignalP-6.0/, accessed on 17 December 2023) [46]. CAMP_R3_ (http://www.camp3.bicnirrh.res.in/, accessed on 7 January 2024) is a database of sequences, structures, and family-specific signatures of prokaryotic and eukaryotic AMPs, and four machine-learning —support vector machine (SVM), discriminant analysis (DA), artificial neural network (ANN), and random forest (RF)— methods from the AMP prediction tool (http://www.camp3.bicnirrh.res.in/prediction.php, accessed on 7 January 2024) were used to *in silico* analyze the mature and active peptide regions of all the contigs annotated as antimicrobial [49]. The computed minimum threshold for a sequence to be regarded as antibacterial is 0.5. The algorithms were applied to all the sequences, and those with scores greater than 0.5 were automatically categorized by the software as putative antimicrobials. Antifp (https://webs.iiitd.edu.in/raghava/antifp, accessed on 7 January 2024) was developed to predict and design antifungal peptides; this server outputs the result as a numerical score, and 0.5 was used as the threshold criterion for this investigation [50]. The iACP web server (http://lin.uestc.edu.cn/server/iACP, accessed on 7 January 2024) has been used to identify the peptides with anticancer activity, based purely on its sequence information alone and via anticancer and non-anticancer scores [51]. The prediction of the antiviral activity was performed with the online server AVPpred (http://crdd.osdd.net/servers/avppred/, accessed on 7 January 2024), whose prediction is based on antiviral peptide motifs (with an output of YES or NO), sequence alignment, amino acid compositions, and physiochemical properties. It provides an overall prediction that is expressed with a cumulative prediction by 0, 1, 2, 3 or by all 4 methods [52].

### 2.6. Evaluation of the Physicochemical Properties

The Antimicrobial Peptide Database Calculator and Predictor tool (https://aps.unmc.edu/prediction, accessed on 7 January 2024) on the antimicrobial peptide database (APD3) [53,54] and the Compute pI/Mw tool—Expasy [47,55,56,57] were used to determine the corresponding physicochemical properties of the putatively identified active peptides after *in silico* analysis, such as peptide length, molecular weight, total hydrophobic ratio, total net charge, isoelectric point, and the Boman index.

### 2.7. Evolutionary Relationships of AMPs

The AMP sequences were correctly annotated and mapped to the *R. ferrugineus* genome [58] (GenBank accession numbers GCA_014462685.1 and GCA_014490705.1) using a BLASTn search against the *R. ferrugineus* genome, which was created using Geneious v7.1.9 (Biomatters) and correctly annotated. The exon-intron positions of the AMPs in the genome were mapped at the scaffold region in a different locus [58]. The mapped regions were extracted and manually aligned using the MAFFT program v7 38, which was used for gene structure illustrations. The NCBI graphical sequence viewer (v 3.50.0), available in the NCBI Genome Workbench, was used to graphically display the nucleotide and protein sequences at the scaffold region in a different locus. The amino acid sequence similarity of AMP genes was tested with the Psi-BLAST (NCBI) sequence alignment algorithm, based on e-value, bit-score, and percent identity. An attempt to infer duplication events through unrooted protein trees for the AMPs produced using BLAST pairwise alignment in the NCBI Tree Viewer (v 7.1.0.46) was performed. The AMP protein sequence similarity search using BLASTp e-value with a cutoff expectation of <2 and <10-e3 identified different AMP clades, shedding light on gene duplication events.

## 3. Results

### 3.1. De Novo Transcriptome Assembly and Gene Identification

To clearly identify the putative peptide candidates, next-generation sequencing (NGS) RNAseq was performed on the RNA isolated from the transcriptomes of male and female *R. ferrugineus* adults from different body parts: abdomen, antennae, fat body, gut, head, legs, snout, thorax, and wings. Specimens were collected from both laboratory and field environments. The raw data have been deposited in the National Center for Biotechnology Information (NCBI) Sequence Read Archive (SRA) database, with the accession numbers for the different body parts of the red palm weevil detailed in Supplementary File S1.

NGS performed with RNA isolated from the abdomen, fat body, thorax, antennae, gut, head, legs, snout, and wings of *R. ferrugineus* male and female adults, bred in the lab or collected in the field, allowed us to generate a *de novo* transcriptome assembly (Table 2). All sequences were subjected to gene ontology (GO) analysis in Blast2GO for functional annotation (Table 2).

Annotation of the *de novo* assembly of the adult transcriptomes led to the identification of 827 contigs, which were annotated as putative antimicrobial sequences and subsequently analyzed.

### 3.2. Alignment of Sequences

This work used the coloring methods for multiple sequence alignment from the Clustal Omega tool. This approach allowed for the alignment of multiple sequences, highlighting areas of similarity that may be associated with specific features conserved more highly than in other regions (Supplementary File S2). Residues were colored according to their physicochemical properties, as reported in Table 1. The alignment was also useful for detecting duplicate contigs. In total, 457 duplicated sequences were detected in various body parts. Specifically, the field male duplicates were predominantly expressed in the fat body, the laboratory male duplicates in the head, the field female duplicates in the wings, and the laboratory female duplicates in the abdomen and legs.

Regarding duplicates (repeated sequences) in individual parts of the body, the following were identified: 5 duplicates in the abdomen, all of which were expressed in field females; 5 duplicates in the fat body, mostly identified in males in an equal number of field and laboratory males; 10 duplicates in the head, mostly expressed in laboratory males; 13 duplicates in the snout, mostly expressed in field males; 13 duplicates in the antennae, mostly expressed in field females; 5 in the gut, mostly expressed in field males; 14 in the legs in an equal number of field females and field males; 13 in the thorax, mostly in field females; and 18 in the wings, mostly in field females.

### 3.3. Expression Levels of Duplicate AMP Genes

**Figure 1:**
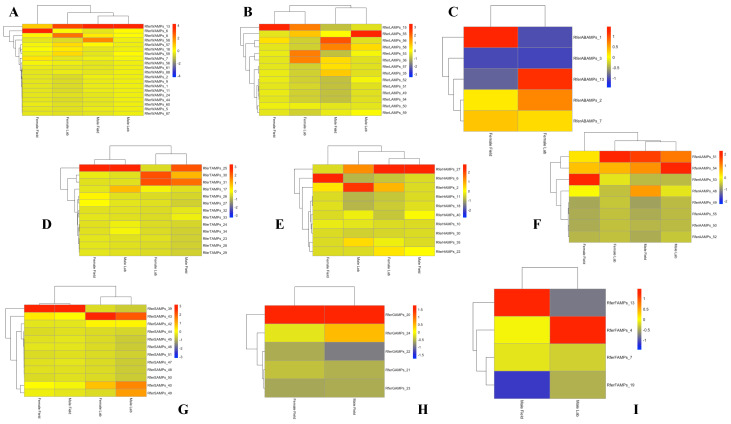
Heatmap showing the expression levels of duplicated genes in the different body parts of *Rhynchophorus ferrugineus* lab-reared and field-collected male and female adults. The heatmap colors represent transcript abundance in transcripts per million (TPM) from highest (red) to lowest (blue) expression levels. The data represented as log-transformed TPM values were tabulated and converted into heatmaps using R and R Studio software (version 2023.06.2+561). (**A**) Wings; (**B**) Legs; (**C**) Abdomen; (**D**) Thorax; (**E**) Head; (**F**) Antennae; (**G**) Snout; (**H**) Gut; (**I**) Fat Body.

Following alignment, the TPM (transcripts per million) values of duplicated sequences were compared (Supplementary File S3) in order to evaluate the gene expression levels of AMPs in each part of the body in different experimental conditions. The same sequences showed different expression levels, as reported in the heat maps (Figure 1).

### 3.4. Antimicrobial Activity Prediction

The sequences of interest detected in the adult transcriptome were analyzed using the free online database CAMP_R3_ to predict their putative antimicrobial activity. According to the antimicrobial software analysis, 198 sequences showed putative antimicrobial activity.

**Figure 2:**
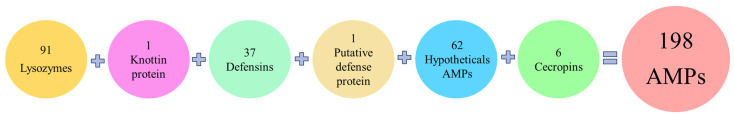
Classification of the 198 total AMPs detected in adult transcriptomes related to their family classification.

Among the 198 genes encoding putative AMPs in the *R. ferrugineus* adult transcriptomes were identified 37 defensins, 6 cecropins, 62 hypothetical antimicrobial peptides, 1 knottin protein, 1 putative defense protein, and 91 between lysozymes and lysozyme-like proteins (Figure 2). Although lysozymes are not typically classified as AMPs due to their large size, they were taken into consideration because of their known antimicrobial properties.

### 3.5. Antifungal, Anticancer, and Antiviral Activity Prediction

The 198 sequences were also analyzed to predict their antifungal, anticancer, and antiviral activity.

From Supplementary File S4, it is evident that five AMPs exhibit putative antifungal activity, while 101 contigs with putative anticancer activity were identified. However, none were found to have putative antiviral activity. Among the five AMPs with putative antifungal activity, two defensins and three lysozymes were identified. Among the 101 AMPs with putative anticancer activity, 23 are defensins, 54 are lysozymes or lysozyme-like, 20 are hypothetical antimicrobial peptides, 1 is a putative defense protein and 3 are cecropins (Supplementary File S4).

Specifically, these 198 AMPs displayed various combinations of antimicrobial, anticancer, and antifungal activity. Additionally, four peptides were found to possess all three activities: two defensins, and two lysozymes (Supplementary File S4).

### 3.6. Physicochemical Properties of the Identified Peptides

The physicochemical properties (length in amino acid residues, molecular weight (MW), total hydrophobic ratio in percentage, total net charge, isoelectric point (pI), and the Boman index in kcal/mol) of the putative peptides have been identified using the Antimicrobial Peptide Database Calculator and Predictor (APD3) and the Compute pI/Mw tool—Expasy and are reported in Supplementary File S4.

In particular, the length of amino acid residues in the sequences ranges from a minimum of 20 residues to a maximum of 165 residues in the adult transcriptomes (Supplementary File S4).

Most peptides with a low number of amino acid residues, a low hydrophobicity index, and a cationic nature are defensins. The two defensins with putative antifungal, antimicrobial, and anticancer activity have 44 and 43 amino acid residues, respectively, and very similar total hydrophobic ratios and positive total net charges. While there is no definitive criterion for correlating predicted physicochemical properties with corresponding bioactivities across all sequences, a notable association exists for most of them. Specifically, there is an interesting link between positive charge and putative antimicrobial activity, with 158 out of 198 sequences exhibiting this characteristic.

### 3.7. Evolutionary Relationships of AMPs

**Figure 3:**
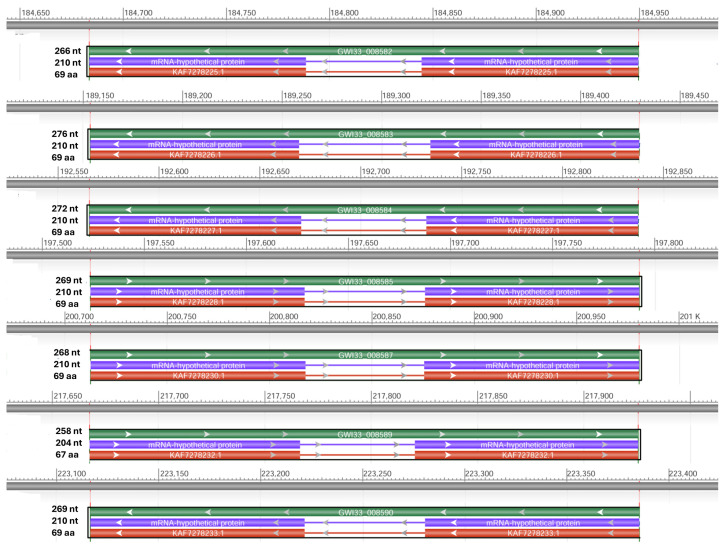
Graphical display for the cecropin genes mapped in scaffold_405 (NCBI acc no. JAACXV010000404.1) showing functional cecropins gene length, CDS (coding region) length, and protein length, which was generated using the NCBI graphical sequence viewer available in the Genome workbench. The visual code shows green, red, and purple, indicating gene, coding region, and mRNA, respectively. The line shows the introns and boxes for the exons.

By using NCBI Acc. No. JAACXV010000404.1, we annotated and mapped all seven cecropins fragments in scaffold_405 onto the *R. ferrugineus* genome. The genomic organization revealed that the five distinct cecropins genes were distributed in locus_tag = “GWI33_008582, GWI33_008583, GWI33_008584, GWI33_008585, GWI33_008587, GWI33_008589 and GWI33_008590” and then across the same scaffold_405 in the *R. ferrugineus* genome, indicating possible emergence through tandem duplication (Figure 3). The functional cecropins gene length, CDS (coding region) length, and protein length mapped in scaffold_405 are shown in Figure 3. All cecropin genes contained two exons and an intron (Figure 3).

**Figure 4:**
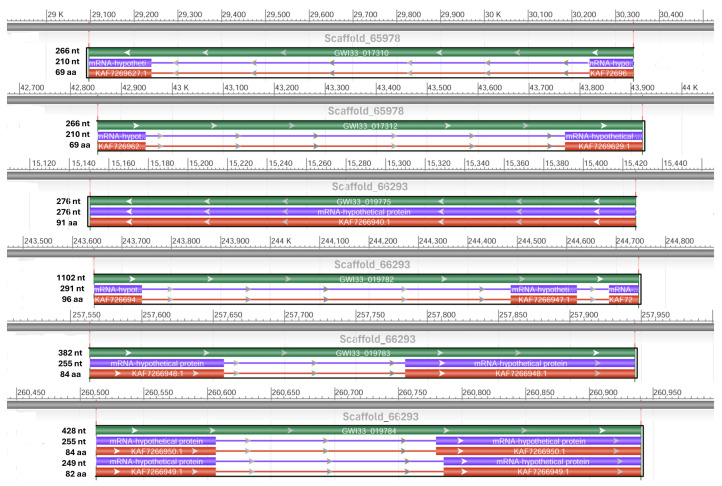
Graphical display for the defensin genes mapped in the six scaffolds (NCBI acc no. JAACXV010014362.1, JAACXV010000413.1, JAACXV010014484.1, JAACXV010014200.1, JAACXV010014575.1, and JAACXV010014362.1) showing functional gene length, CDS length, and protein length generated using the NCBI graphical sequence viewer available in the Genome workbench. The visual code shows green, red, and purple, indicating gene, coding region, and mRNA.

Using NCBI DBSOURCE accessions JAACXV010014362.1, JAACXV010000413.1, JAACXV010014484.1, JAACXV010014200.1, JAACXV010014575.1, and JAACXV010014362.1, we annotated a deduced amino acid sequence of the six distinct defensin genes. Six scaffolds have been identified as carrying at least one, and often more than one, defensin gene. The genomic organization of defensins revealed that they were distributed across different scaffolds in the *R. ferrugineus* genome with an uneven distribution pattern (Figure 4). Among these, the first four major defensin genes were represented in the locus tags: GWI33_018859 (scaffold_66293), GWI33_009171 (scaffold_414), GWI33_020600 (scaffold_66394), and GWI33_018859 (scaffold_66154). Among the last two remaining defensins, there were four duplicates (locus_tag = “GWI33_019775, GWI33_019782, GWI33_019783, and GWI33_019784”) distributed across the same scaffold_66293 (JAACXV010014484.1), and other two duplicates (locus_tag = “GWI33_017310 and GWI33_017312”) in the same scaffold_65978 (JAACXV010014200.1) in the *R. ferrugineus* genome, indicating possible emergence through tandem duplication (Figure 4).

**Figure 5:**
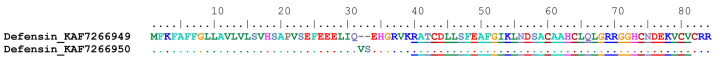
Two allelic variants in the locus tag “GWI33_019784” and deduced amino acids predict 82aa and 84-aa proteins (NCBI acc nos. KAF7266949 and KAF7266950), with a conserved DEFL_defensin-like domain at the C-terminal region. Dots denote identical amino acid residues, and conserved DEFL_defensin-like domain at the C-terminal region are underlined.

We identified two allelic variants in the locus tag “GWI33_019784” and the deduced amino acids predict 82-aa and 84-aa proteins (NCBI acc nos. KAF7266949 and KAF7266950), with a conserved DEFL_defensin-like domain at the C-terminal region (Figure 5). The amino acid sequence of both alleles has ≈ 98% identity, and two amino acids (Val32 and Ser33) were deleted in the defensin acc no. KAF7266949 (Figure 5). The functional defensin gene length, CDS length, and protein length mapped in scaffold_66293 and scaffold_65978 are shown in Figure 4.

**Figure 6:**
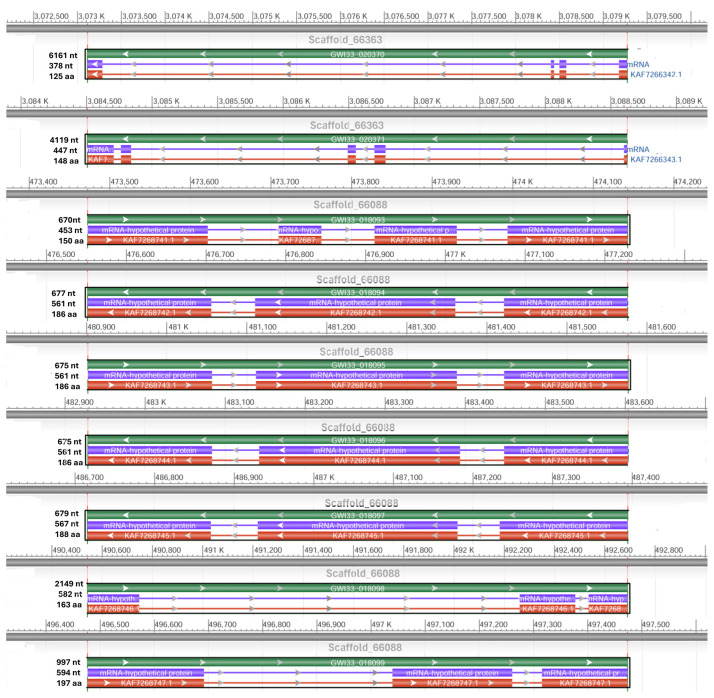
Graphical display for the hypothetical AMP genes mapped in the two scaffolds (66363 and 66088) (NCBI acc nos. JAACXV010014549.1 and JAACXV010014301.1) showing the functional AMP gene length, CDS length, and protein length generated using the NCBI graphical sequence viewer available in the Genome workbench. The visual code shows green, red, and purple, indicating gene, coding region, and mRNA, respectively. The line shows the introns and boxes for the exons.

We retrieved two scaffolds (66363 and 66088) containing potential hypothetical AMPs and, using NCBI DBSOURCE accessions JAACXV010014549.1 and JAACXV010014301.1, we annotated a deduced amino acid sequence for the two major hypothetical AMP families. The first group of hypothetical AMPs in the same scaffold_66363 (JAACXV010014549.1) contains two genes and was mapped in the locus tags GWI33_020371 and GWI33_020371 in the *R. ferrugineus* genome, indicating possible emergence through tandem duplication (Figure 6). The second group of hypothetical AMPs mapped in the scaffold_66088 (JAACXV010014301.1) has seven distinct tandem duplicates that were distributed in the locus tags = “GWI33_018093, GWI33_018094, GWI33_018095, GWI33_018096, GWI33_018097, GWI33_018098 and GWI33_018099” (Figure 6). The functional AMP gene length, CDS length, and protein length mapped in scaffold_66363 and scaffold_66088 are shown in Figure 6.

**Figure 7:**
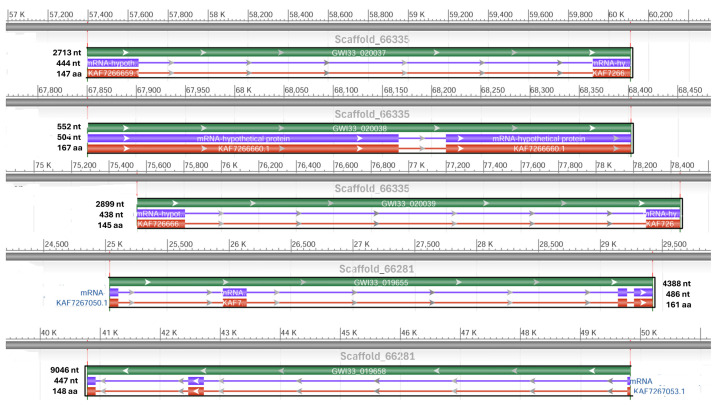
Graphical display for the lysozyme genes mapped in the two scaffolds (66335 and 66281) (NCBI acc nos. JAACXV010014523.1 and JAACXV010014472.1) showing functional lysozyme gene length, CDS length, and protein length generated using the NCBI graphical sequence viewer available in the Genome workbench. The visual code shows green, red, and purple, indicating gene, coding region, and mRNA, respectively. The line shows the introns and boxes for the exons.

We retrieved six scaffolds that have been identified as carrying at least one, and often more than one, lysozyme gene using NCBI DBSOURCE accessions JAACXV010014523.1, JAACXV010014472.1, JAACXV010000242.1, JAACXV010000047.1, JAACXV010013077.1, and JAACXV010013977.1. The first group of lysozymes in the same scaffold_66335 contained three distinct genes. It was mapped in the locus tags GWI33_020037, GWI33_020038, and GWI33_020039 (JAACXV010014523.1) in the *R. ferrugineus* genome, indicating possible emergence through tandem duplication (Figure 7). The second group of putative lysozymes mapped in the scaffold_66281 (JAACXV010014472.1) had two distinct tandem duplicates that were distributed in locus_tags = “GWI33_019655 and GWI33_019658. The functional AMP gene length, CDS length, and protein length mapped in scaffold_66335 and scaffold_66281 are shown in Figure 7. The remaining four lysozymes were distributed in the scaffold_243 (JAACXV010000242.1), scaffold_48 (JAACXV010000047.1), scaffold_64653 (JAACXV010013077.1), scaffold_65725 (JAACXV010013977.1), and mapped in the locus tags: GWI33_004805, GWI33_010182, GWI33_013206 and GWI33_015786 respectively. All these lysozymes were distinct genes with no evidence of duplication event.

## 4. Discussion

The alarming spread of multidrug-resistant infections has prompted researchers to search for new antibacterial substances [59]. AMPs are promising candidates as alternatives to conventional antibiotics due to their low toxicity to eukaryotic cells and their broad spectrum of action against bacteria, mycobacteria, fungi, viruses, and cancer cells [60]. AMPs can kill bacteria in various ways, such as by disrupting membranes, concentrating on intracellular components, or interfering with metabolism [61,62]. Most AMPs are also cationic, which enhances their ability to interact electrostatically with negatively charged bacterial membranes [63,64]. Because of their enormous biodiversity and varied living conditions, insects are among the richest sources of AMPs among all living organisms [65,66].

In this study, we examined the putative genes encoding AMPs in the transcriptomes of *R. ferrugineus*, along with their expression levels and potential functions.

Multiple sequence alignments facilitated the detection of duplicate sequences. Alignments performed with Clustal Omega software were useful in identifying duplicate sequences within each body part and across different parts of the adult transcriptomes, resulting in the identification of 457 duplicated sequences in total.

The occurrence of duplicate transcripts may be attributed to gene duplication events. Specifically, the genome analysis of RPW revealed the existence of 837 duplicate genes, some of which are involved in pesticide resistance or detoxification processes [67]. Gene duplication is widely recognized as a primary mechanism contributing to the emergence of structural and functional diversity during genome development [68]. Moreover, it is a widespread phenomenon for AMPs. An adaptive model predicts that duplicated genes have distinct expression patterns [69], as it is possible to appreciate in this work.

After detecting the duplicate sequences, we evaluated the expression level of these sequences by comparing RPKM (reads per kilobase of exon per million mapped reads) values. This metric quantifies the relative abundance of a transcript within a population of sequenced transcripts, firstly in every individual part of the body and then between each part of the body of the adults.

The remaining sequences were analyzed *in silico* using the CAMP_R3_ database to evaluate their antimicrobial activity; a total of 198 sequences resulted in a putative antimicrobial activity. These sequences were analyzed by bioinformatic tools to detect and classify the putative activity of each peptide. It is possible to determine, therefore, whether a certain sequence corresponds to a peptide that acts, at least *in silico*, against viruses, bacteria, fungi and/or cancer cells [70,71,72].

Using databases to identify the similarities among the sequences with antimicrobial, antiviral, antifungal, and antitumor activity allows for the rapid identification of potentially valid sequences, facilitating a deeper exploration of these studies [73]. The bioinformatics approach, therefore, represents a valid starting point in the study of sequences present in transcriptomes, where there is a huge amount of data [74]. This allows a preliminary but efficient screening for selecting the most promising candidates that can then be progressively produced (through chemical synthesis or recombinant DNA technology) and functionally characterized [65,75,76].

A similar *in silico* approach was adopted in the study of Moretta et al. [56], where the larvae and the combined adult male and female *Hermetia illucens* transcriptomes were examined. This identified 57 putatively active peptides, 13 of which with antimicrobial activity, 22 with antimicrobial and anticancer activity, 8 with antimicrobial and antiviral activity, 2 with antimicrobial and antifungal activity, 7 with antimicrobial, anticancer and antiviral activity, 1 with antimicrobial, antiviral and antifungal activity, 2 peptides with antimicrobial, anticancer and antifungal activity, and another 2 positive to all activity predictions [65]. Five putative AMPs, which showed high antimicrobial scores with all prediction algorithms, were selected, chemically synthesized, or produced by recombinant DNA technology and positively screened against *E. coli*, *S. aureus,* and *Staphylococcus epidermidis*, confirming the antimicrobial activity evaluated *in silico* [7,65]. Data from *H. illucens* transcriptomes were also the starting point for a combined approach (transcriptomic and proteomic) to identify AMPs expressed in haemolymph under different experimental conditions [77].

In the work of Li et al. [76], defensins of the insect *Periplaneta americana* were identified by comparing the genome sequences of the insect with AMP sequences in databases. The authors identified five putative defensins and studied their physicochemical characteristics (length, amino acid composition, molecular weight, total hydrophobic ratio, total net charge, and isoelectric point), as well as their primary and secondary structures and the putative antimicrobial, antifungal and antiviral activity. Two AMPs demonstrated both antimicrobial and antiviral activity, while the other three defensins were predicted to have exclusively antiviral activity. The authors directed further experiments on AMPs with exclusive antiviral activity, conducting tests on *Drosophila melanogaster* Kc cells infected with the *Drosophila* C virus [76]. The viral DNA expression levels showed a reduced virus titer in cells treated individually with all three peptides. The most promising defensin was used in further experiments, showing a positive impact in *D. melanogaster* Kc cells by preventing apoptosis induced by viral infection, and in *D. melanogaster* larvae, limiting infection and increasing survival rate. These results confirmed the bioinformatics predictions.

The study of RPW transcripts, obtained under experimental conditions through *in silico* methods, has facilitated the identification of a significant number of AMPs, primarily falling into the defensins, cecropins, knottin proteins, and lysozymes classes. Defensins are used as a defense mechanism in various insect orders, including Diptera, Hymenoptera, Hemiptera, Coleoptera, and Lepidoptera [13]. Additionally, defensins have been identified in the ancestral order of Odonata, suggesting a potential evolutionary origin of insect defensins from a shared ancestral gene. Defensins are rich in cationic arginine residues; they comprise 6–8 preserved cysteine residues and vary in size from 18 to 45 amino acids, and insect defenses are usually composed of 29–35 amino [78]. The fundamental structural feature of the defensin molecule is a β-hairpin, which is typically stabilized by three disulfide bonds [79]. Due to the outflow of vital and nutritious ions from the cell, the defensins bind to the cell membrane or create pore-like membrane defects [25]. Insect defensins primarily exhibit antimicrobial activity against Gram-positive bacteria, such as *Micrococcus luteus*, *Aerococcus viridians*, *Bacillus megaterium*, *Bacillus subtilis, B. thuringiensis*, and *S. aureus*. Certain insect defensins have also demonstrated activity against the Gram-negative *E. coli* bacteria and certain fungi [66]. The study conducted by Robles-Fort et al. demonstrates that defensins exhibit a significant antiproliferative effect on triple-negative breast cancer cells while showing negligible cytotoxicity toward normal cells [80].

Cecropins, which play a significant role in the cell-free immunity of insects [26], were initially extracted from the hemolymph of infected pupae of the moth species *H. cecropia*. They have since been identified in various lepidopteran and dipteran species. They are small proteins, with approximately 35 amino acid residues, that have efficacy against Gram-positive and Gram-negative bacteria due to their ability to lyse bacterial cellular membranes, impede proline uptake, and create leaky membranes [27,81]. A, B, and D are the three main insect cecropins comprising 5–37 residues [82]. Henao Arias et al. examined the cecropins derived from *O. curvicornis* and dung beetles [83]. The researchers observed a significant inhibitory effect of these cecropins against Gram-negative bacteria, specifically *E. coli* and *Pseudomonas aeruginosa* [83]. Research investigations on *Drosophila* specimens exhibiting gene deletions responsible for encoding cecropins have substantiated their involvement in the defense mechanism against Gram-negative bacteria, specifically *Enterobacter cloacae* and *Providencia heimbachae* [84]. One of the oldest and most well-known cecropins, Cecropin A, exhibited fungicidal properties against *Candida albicans*, *Malassezia obtuse*, and *Malassezia sloofiae*, which can cause a variety of pathological conditions of mammalian skin, and under certain conditions, antibacterial properties against multidrug-resistant bacteria, both Gram-positive and -negative [85,86]. *In vivo* investigations using C57BL/6 mice that evaluated the impact of cecropin A on induced inflammatory bowel disease (IBD) and alterations in gut microbiota in comparison to gentamicin have also been conducted [87]. In the study of Xu et al., the antitumor activity of *B. mori* AMPs, Cecropin A and Cecropin D, was shown against human esophageal cancer cells, with suppression of tumor cell proliferation, which induced apoptosis and inhibited migration and invasion, and no inhibitory effect on normal human embryonic kidney 293T cells [88].

Lysozymes, another component of the defense mechanism against bacteria, are bacteriolytic enzymes that hydrolyze b-1,4-glycosidic bonds between N-acetylglucosamine and N-acetylmuramic acid of the peptidoglycan layer in the bacterial cell walls [89]. They have been identified in the majority of animals and discovered in the salivary glands and guts of many insects [90,91,92,93]. As in vertebrates, lysozymes in insects are widely distributed as one of the components of bactericidal proteins and peptides found in the hemolymph, and synthesis is induced by infection with bacteria [1].

In this study, the analyses revealed variations in the number of AMPs across different developmental stages of *R. ferrugineus*, including the embryonic stage (E1-SRR926614, E2-SRR926615, E3-SRR926616, E4-SRR926617), larval stage (L1-SRR926618), pupal stage (SRR13297420), and adult stage. Adult transcripts showed a more significant number of contigs coding for putative AMPs compared to the larval transcripts of the same insect. The induction of immunity-related gene expression in RPW larvae is attributed to infection by pathogenic bacteria [94]. It is widely recognized that RPW larvae typically develop within the central region of palm trees, consuming tender tissues and sap, ultimately leading to the demise of the host plant by damaging the meristem [33]. Consequently, it is plausible to associate the reduced expression of AMPs in larvae with their decreased vulnerability to pathogens as a result of the aforementioned behavior. This finding could be supported by Wang et al., whose research revealed that the pupal stage had the greatest overall expression levels of the scale gene, compared to other life stages [37]. During the pupal stage, the morphology and mechanism of the RPW undergo significant alterations.

This concept was also supported by Manee et al. [95], who identified developmental stage-associated genes, differentially expressed genes, conserved signaling pathways, and constitutively expressed genes in RPW. Furthermore, in Yang et al. [96], the gene expression values of different developmental stages of RPW were analyzed, and the results indicated that pupae and male adults have an overall higher expression level. These findings supported the biological differences between the larval, pupal, and adult development stages of *R. ferrugineus* [96].

The higher presence of AMPs in pupae, compared to larvae, may be attributed to the need for protection during this transitional phase of life, characterized by tissue changes and reorganization [97,98,99]. In some instances, stronger immune activity has been noted during larval and adult stages [100]. However, comparisons between different species are challenging, as numerous factors can influence immune system response and AMP production.

Differences in antimicrobial response were experimentally tested in Cappa et al. [32], and Mazza et al. [101]. In these studies, larvae showed higher mortality compared to adults, and egg extracts could not stop the bacterial growth. Even though the eggs live in the same habitat as larvae and adults, they quickly hatch, and the chorion physical barrier may shield them from microbial attack [101].

The presence of AMPs in various body parts is also supported by Ma et al. [94], whose study showed significantly different expression levels of specific proteins in multiple tissues, such as the hemolymph, foregut, midgut and hindgut, fat body, head, and epidermis of RPW larvae. In particular, the gut and fat body are interesting due to their roles in the local immunocompetence of RPW larvae and the modulation of RPW immune homeostasis. Additionally, Muhammad et al. verified that the immune system of RPW plays a central role in sustaining and modulating the homeostasis of gut commensal microbiota [102]. Their study found that the presence of gut microbiota strongly upregulated the immunity-related genes of RPW larvae, which contain pathogen recognition receptors, nuclear factors, and in particular, AMPs. The study also revealed that the presence of gut bacteria could significantly improve the transcript abundance of several pattern recognition receptors (PRRs) and antimicrobial peptides also in the fat body [102]. These findings provide experimental evidence to support the conclusion that colonization of gut commensal microbiota has stimulatory effects on the immune system of RPW larvae, enhancing the host’s immunocompetence through the upregulation of immune genes, which in turn stimulates the secretion of AMPs. Several studies have revealed that the gut microbiome can inhibit the development of *Plasmodium* and other human pathogens in mosquitos by upregulating important immune genes such as cecropins, defensins, and gambicins [103,104].

AMPs also play an important role in insect legs. According to Nomura et al. [105], regenectin, a C-type lectin found in hemolymph, appears transiently around developing muscle cells in the late stage of leg regeneration of *P. americana.* This suggests a systemic process that may involve the production of specific AMPs. In light of this, our analysis emphasizes the presence of repeated sequences in particular parts of the body, such as the legs and wings. This is probably because, in the adult, these peptides are crucial for properly supporting muscle development and regeneration of the legs and wing apparatus, which are fully sclerified and particularly robust in the adults of Coleoptera [105,106].

We manually annotated AMPs in the *R. ferrugineus* genome [58] and these annotations allowed us to correct the previous transcriptome annotation. Genome (GenBank: GCA_014462685.1) analyses revealed several AMP duplications from two to seven genes in clusters in the same scaffold. Of particular interest are seven cecropin genes in the cluster on scaffold_405, four defensin genes in the cluster on scaffold_66293, seven hypothetical AMP genes in the cluster in scaffold_66088, and three lysozyme genes in the cluster on the scaffold_66335, all representing potentially recent duplications. AMP gene duplications are of major interest since insect AMPs are known to evolve rapidly through gene gain and loss, followed by gene loss, divergence, and other processes, allowing each species to adapt to its specific ecological niche [107]. Elucidating the function of the different AMP duplicates would allow us to assess whether duplicates retained similar functions or evolved different functions. Interestingly, we identified two allelic variants of defensin in the locus tag GWI33_019784, which shared 99% identity with two amino acids (Val32 and Ser33) that were deleted in the defensin in one allele (Figure 5). As defensins exhibit a diversity of antimicrobial mechanisms, such as direct membrane disruption, neutralizing microbial toxins, and inhibition of microbial cell wall synthesis [108], the allelic variation in a defensin gene essential for these multifunctional roles accounts for the functional variations, which is an exciting area of further research.

## 5. Conclusions

This work analyzed *R. ferrugineus* transcriptomes data from adult females and males reared in laboratory and field conditions, using different bioinformatic tools to predict putative antimicrobial, antifungal, and anticancer activities.

In conclusion, although the results of the computational approach must be confirmed *in vitro* and *in vivo*, this strategy allows us to narrow the search field and select the most promising molecules from the growing amount of available data, thus supporting and expediting the discovery of new drugs.

## Figures and Tables

**Table 1:** Criteria of multiple alignments. The residues of the aligned sequences are coded according to the following criteria: AVFPMILW is shown in red, DE in blue, RHK in magenta, STYHCNGQ in green, and all other residues in grey. The residue range for each sequence is shown after the sequence name.

Residue	Color	Property
AVFPMILW	RED	Small (small + hydrophobic (including aromatic − Y))
DE	BLUE	Acidic
RHK	MAGENTA	Basic − H
STYHCNGQ	GREEN	Hydroxyl + sulfhydryl + amine + G
Others	GREY	Unusual amino/imino acids etc.

**Table 2:** List of analyzed transcriptomes. From left to right the name of the transcriptome, total contigs, minimum length, maximum length, % of contigs matching with assigned molecular functions in the GO database, and % of contigs non-matching with assigned molecular functions in the GO database are shown.

Transcriptome	Total Contig	Min Length (bp)	Max Length (bp)	% of Contigs Matching with Assigned Molecular Functions in the GO Database	% of Contigs Non-Matching with Assigned Molecular Functions in the GO Database
Abdomen Female Field	24.564	125	8.417	73	27
Abdomen Female Lab	27.961	106	8.383	68	32
Fat Body Male Field	38.109	107	17.994	56	44
Fat Body Male Lab	38.226	108	11.542	61	39
Thorax Female Field	24.289	97	11.037	74	26
Thorax Female Lab	28.064	69	5.700	69	31
Thorax Male Field	30.708	100	12.801	64	36
Thorax Male Lab	25.211	100	4.822	74	26
Antennae Female Lab	81.862	101	21.384	46	54
Antennae Female Field	59.627	104	25.370	47	53
Antennae Male Field	53.645	74	21.051	52	48
Antennae Male Lab	47.910	89	14.929	61	39
Gut Female Field	67.747	87	25.094	44	56
Gut Male Field	31.308	92	14.443	61	39
Head Female Field	28.195	125	22.558	72	28
Head Female Lab	35.232	107	16.119	66	34
Head Male Field	30.748	125	13.320	68	32
Head Male Lab	35.527	90	12.875	66	34
Legs Female Field	68.240	83	38.106	58	42
Legs Female Lab	70.207	115	12.319	58	42
Legs Male Field	75.700	101	23.454	57	43
Legs Male Lab	105.309	102	23.303	52	48
Snout Female Field	63.109	121	30.807	50	50
Snout Female Lab	120.977	72	21.750	93	7
Snout Male Field	126.195	104	34.191	52	48
Snout Male Lab	62.512	106	13.318	59	41
Wings Female Field	72.889	94	18.328	52	48
Wings Female Lab	46.395	115	17.855	58	42
Wings Male Field	73.685	93	19.966	53	47
Wings Male Lab	38.804	123	25.527	58	42

## Data Availability

The datasets used and/or analyzed in this study are available from the corresponding author upon reasonable request. The RPW transcriptomes (male and female; laboratory and field) have been deposited in NCBI/DDBJ/EMBL SRA accession numbers BioProject PRJNA275430) and are reported in Supplementary File S1.

## Supplementary Materials

The following supporting information can be downloaded at: https://www.mdpi.com/article/10.3390/biom14101332/s1, Supplementary File S1: NCBI Accession numbers for different body parts of the Red Palm Weevil (*Rhynchophorus ferrugineus*). Data are collected from male and female specimens under both laboratory and field conditions, as reported in the SRA. Supplementary File S2: Multiple sequence alignments using coloring methods (Clustal Omega) of the peptide sequences from all parts of the body of male and female *R. ferrugineus* adult, both reared in laboratory and collected in field. Supplementary File S3: Expression Levels of Duplicate AMP Genes comparing the TPM (transcripts per million) values of duplicated sequences to evaluate the gene expression levels of putative antimicrobial sequences in each part of the body in different experimental conditions. Supplementary File S4: List of the 198 putative AMPs, identified in the transcriptome of the different body part of male and female *R. ferrugineus* adult, both reared in laboratory and collected in field. In the table are reported: sequence name, contig name and number, the adult condition (lab or field), the adult sex (male or female), the sequence description (annotation), the mature amino acid sequence, results of the antimicrobial (CAMP_R3_), anticancer (iACP), antifungal (ANTIFP) antiviral (AVPpred), physicochemical parameter (APD3) (length, molecular weight, total hydrophobic ratio, total net charge, pI, Boman Index).
