# Supplementary material for: Identification of Multifunctional Putative Bioactive Peptides in the Insect Model Red Palm Weevil (Rhynchophorus ferrugineus)
Source: Biomolecules. 2024 Oct 19;14(10):1332. doi: 10.3390/biom14101332 (PMC11506011; doi:10.3390/biom14101332)
Supplement: Supplementary file 1 [file biomolecules-14-01332-s001.zip › biomolecules-3172528_Supplementary File S1.pdf]

	SRA			
**Part of the body**	**Male Laboratory**	**Male Field**	**Female Laboratory**	**Female Field**
Abdomen	-	-	SRR27695093	SRR27695094
Antennae	SRR22098126	SRR22098127	SRR22098128	SRR22098129
Fat body	SRR29346546	SRR29346547	-	
Head	SRR29346787	SRR29346788	SRR29346789	SRR29346790
Gut	-	SRR27695095	-	SRR27695096
Legs	SRR29221206	SRR29221207	SRR29221208	SRR29221209
Snout	SRR17732026	SRR17732027	SRR17732028	SRR17732029
Thorax	SRR29346791	SRR29346792	SRR29346793	SRR29346794
Wings	SRR29346548	SRR29346549	SRR29346550	SRR29346551

Supplementary File S1 NCBI Accession numbers for different body parts of the Red Palm Weevil (*Rhynchophorus ferrugineus*). Data are collected from male and female specimens under both laboratory and field conditions, as reported in the SRA**.**
