# Supplementary material for: Identification of Multifunctional Putative Bioactive Peptides in the Insect Model Red Palm Weevil (Rhynchophorus ferrugineus)
Source: Biomolecules. 2024 Oct 19;14(10):1332. doi: 10.3390/biom14101332 (PMC11506011; doi:10.3390/biom14101332)
Supplement: Supplementary file 1 [file biomolecules-14-01332-s001.zip › biomolecules-3172528_Supplementary File S2.pdf]

```
head_contig_22785          ----------------FPGLTKHSDEENVYIHPSFVTDTLTD-PH---------------
antennae_contig_18354      ----------------------EEKNNGI-YEEVIV------------------------
gut_contig_34289           ----------------------EEKNNGI-YEEVIV------------------------
legs_contig_18143          ------------------LPYAEEKNNGI-YEEVIV------------------------
snout_contig_25043         ----------------------EEKNNGI-YEEVIV------------------------
wings_contig_17859         ------------------QQYTIEDEEDIYRQDSIV------------------------
snout_contig_8906          ------------------QQYTIEDEEDIYRQDSIV------------------------
snout_contig_6533          ------------------QQYTIEDEEDIYRQDSIV------------------------
fat-body_contig_18846      ------------------QPYPIEDEENAYRQDSIV------------------------
head_contig_1816           -------------------------------------------------------MFKFV
gut_contig_36950           --------------------------------------------------MKLFSVFMLV
gut_contig_19714           --------------------------------------------------MKFLHIFVFV
head_contig_1777           -------------------GVALCYQAGQYVPKSFYI--------IDQFGRKSDLVYVRN
thorax_contig_875          -------------------GVALCYQAGQYVPKSFYI--------IDQFGRKSDLVYVRN
wings_contig_282           ------------------------YQAGQYVPKSFYI--------IDQFGRKSDLVYVRN
legs_contig_679            ------------------------YQAGQYVPKSFYI--------IDQFGRKSDLVYVRN
gut_contig_1217            ------------------------YQAGQYVPKSFYI--------IDQFGRKSDLVYVRN
abdomen_contig_7701        -------------------GVALCYQAGQYVPKSFYI--------IDQFGRKSDLVYVRN
antennae_contig_4063       -------------------GVALCYQAGQYVPKSFYI--------IDQFGRKSDLVYVRN
gut_contig_631             --------------------VALCYQAGQYVPKSFYI--------IDQFGRKSDLVYVRN
snout_contig_872           -------------------GVALCYQAGQYVPKSFYI--------IDQFGRKSDLVYVRN
thorax_contig_5237         -------------------GVALCYQAGQYVPKSFYI--------IDQFGRKSDLVYVRN
legs_contig_17294          ------------------------YQAGPYVPKSFYI--------IGQVGRKSGLGYVRN
wings_contig_264           ------------------------YQAGQYVPKSFYI--------IDQFGRKSDLVYVRN
wings_contig_247           ------------------------YQAGQYVPKSFYI--------IDQFGRKSDLVYVRN
thorax_contig_5238         -------------------GVALCYQAGQYVPKSFYI--------IDQFGRKSDLVYVRN
legs_contig_738            ------------------------YQAGQYVPKSFYI--------IDQFGRKSDLVYVRN
legs_contig_678            ------------------------YQAGQYVPKSFYI--------IDQFGRKSDLVYVRN
legs_contig_236            ------------------------YQAGQYVPKSFYI--------IDQFGRKSDLVYVRN
legs_contig_1006           ------------------------YQAGQYVPKSFYI--------IDQFGRKSDLVYVRN
head_contig_2691           ------------------------YQAGQYVPKSFYI--------IDQFGRKSDLVYVRN
head_contig_1779           ------------------------YQAGQYVPKSFYI--------IDQFGRKSDLVYVRN
gut_contig_630             ------------------------YQAGQYVPKSFYI--------IDQFGRKSDLVYVRN
antennae_contig_5028       ------------------------YQAGQYVPKSFYI--------IDQFGRKSDLVYVRN
antennae_contig_304        ------------------------YQAGQYVPKSFYI--------IDQFGRKSDLVYVRN
abdomen_contig_7702        -------------------GVALCYQAGQYVPKSFYI--------IDQFGRKSDLVYVRN
gut_contig_1216            ------------------------YQAGQYVPKSFYI--------IDQFGRKSDLVYVRN
head_contig_25197          --------------------LVFCYFVFEATAQISVQRQCRK----------NSEAYRRV
snout_contig_3247          --------------------------RTNSIFFSSVQRQCRK----------NSEAYRRV
head_contig_10144          ---------------------------------IIVQRQCRK----------NSEAYRRV
abdomen_contig_1463        ---------------------------------KSVQRQCRK----------NSEAYRRV
head_contig_4692           ----------------------------EDIETKSVQRQCRK----------NSEAYRRV
wings_contig_37004         -----------MMTRKYS------------------------------------------
antennae_contig_20969      -------------------------FPSTWI--QKITK---DAPPYDDD---NSLTKYEV
fat-body_contig_6280       ----------------------------------------------------ITVSCKSA
legs_contig_15521          -----------MYYNKYNKNLSLCNITDVYL--PGNKPNCPATPTYPATTPKFSLALSRT
wings_contig_9480          ----------------G-------------------------------------------
head_contig_21464          -----QVPPGGSWGASL-------------------------------------------
legs_contig_59268          -----------SYDFSG-------------------------------------------
snout_contig_65174         -------NPDGSYDFSG-------------------------------------------
head_contig_7167           ----------------------------------------------------------RV
legs_contig_12571          ----------------------------------------------------------RV
thorax_contig_6557_ORF     ----------------------------------------------------------RV
wings_contig_13576         -------APESVCDDMKPK-----------------------------------------
snout_contig_8907          --------------------------QQY-------------------------------
head_contig_92             -------------------------------------------------------MLTSS
head_contig_1114           -MA------EGR------------------------------------------------
head_contig_1035           GKAEGETKPEGDQDRGQSE-----------------------------------------
fat-body_contig_22298      ---------------MPLSETGQKNRQKFRT--------RR-------------EMFA--
gut_contig_24519           -----------------------------MI--------CS-------------------
gut_contig_34426           ------------------------------------------------------ITFLDR
wings_contig_8642          -------SPEGVAFRMGLKLLDECSRSDFRI--------CMK---------KKAITFLDR
wings_contig_20348         ------------------------------------------------------MIYLEM
thorax_contig_11735_ORF    ----------------------------------------------------QTSVQLKS
snout_contig_10012         ----------------------------------------------------ETTVQLKS
wings_contig_35199         ----------------------------------------------------ETTVQLKS
legs_contig_56079          ---------------------------------MLPVRRL----------RRETTVQLKS
head_contig_20645          ----------------------------------------------------ETTVQLKS
head_contig_22785          ---LQDRFIHDDKN--------------DEDTYMLPVRRL----------RRETTVQLKS
gut_contig_26424           ----------------------------------------------------QTSWQIKN
```

```
snout_contig_1435            ----------------------------------------------------QTSWQIKN
thorax_contig_5468           ----------------------------------------------------QTSWQIKN
snout_contig_1436            ----------------------------------------------------QTSWQIKN
head_contig_18056            ----------------------------------------------------QTSWQIKN
antennae_contig_18354        ----------EDED--------------GQQFYLLPLGRSKR----------QTSWQIKN
gut_contig_34289             ----------EDED--------------GQQFYLLPLGRSKR----------QTSWQIKN
legs_contig_18143            ----------EDED--------------GQQFYLLPLGRSKR----------QTSWQIKN
snout_contig_25043           ----------EDED--------------GQQFYLLPLGRSKR----------QTSWQIKN
wings_contig_10243           ------------------------------------------SEPEKKNTQPQTDWQIRD
legs_contig_25911            ------------------------------------------SEPEKKNTQPQTDWQIRD
abdomen_contig_20364         -----------------------------------------------------TDWQIRD
legs_contig_11863            ------------------------------------------SEPEKKNTQPQTDWQIRD
thorax_contig_12619_ORF      ------------------------------------------SEPEKKNTQPQTDWQIRD
head_contig_3380             ------------------------------------------SEPEKKNTQPQTDWQIRD
snout_contig_16031           ------------------------------------------SEPEKKNTQPQTDWQIRD
gut_contig_12195             ------------------------------------------SEPEKKNTQPQTDWKVES
wings_contig_17859           ----------EDEA--------------GQQYYLVPLSRTKRSEPEKKNTQPQTDWKVES
snout_contig_8906            ----------EDEA--------------GQQYYLVPLSRTKRSEPEKKNTQPQTDWKVES
legs_contig_12933            ------------------------------------------SEPEKKNTQPQTDWKVES
snout_contig_6533            ----------EDEA--------------GQQYYLVPLSRTKRSEPEKKNTQPQTDWKVES
head_contig_12473            ------------------------------------------SEPEKKNTQPQTDWKVES
head_contig_7109             ------------------------------------------SEPAKKNTQAQTDWQIRK
fat-body_contig_18846        ----------EDEE--------------GQQYHLIPLSRSKRSEPAKKNTQAQTDWQIRK
legs_contig_1172             ------------------------------------------SEPAKKNTQAQTDWQIRK
fat-body_contig_15296        -----------------------YCKPLLD------------------------------
fat-body_contig_11849        -----------------------YCKPLLD------------------------------
legs_contig_22292            -------------------------APVEE------------------------------
head_contig_1816             VV--------GL---LIALAVSSHCAPMDE------------------------------
snout_contig_94              -------------------------APMDE------------------------------
snout_contig_4937            -------------------------APVSE------------------------------
snout_contig_809             -------------------------APVEE------------------------------
thorax_contig_10906          -------------------------APVEE------------------------------
head_contig_5676             -------------------------APVSE------------------------------
fat-body_contig_12309        --------------------LSVNSAPVSE------------------------------
fat-body_contig_9117         ----------------------------------------K---------YHWLKKHLKK
wings_contig_11242           ----------------------------------------R---------PSWLKKRLKS
abdomen_contig_11013         -------------------VVAVIFGSTEA----------K---------PSWLKKRLKA
fat-body_contig_4257         ----------------------------------------K---------PSWLKKRLKA
wings_contig_33172           ----------------------------------------K---------PSWLKKRLKA
wings_contig_26325           ----------------------------------------K---------PSWLKKRLKA
thorax_contig_19456          ----------------------TIFVSTEA----------K---------PGWLKKQLKS
gut_contig_36950             -------------------LLVSAAIFNGA----------E---------AGWLKKQLKS
wings_contig_3463            ---------------------------------------------------GWLKKQLKS
head_contig_10176            ------------------LVVATIFGSAEA----------K---------PGWLKKQLKS
head_contig_8472             ----------------------------------------K---------PGWLKKQLKS
wings_contig_19057           ----------------------------------------K---------PGWLKKQLKS
wings_contig_20315           --------------------VATIFGSAEA----------K---------PGWLKKQLKS
thorax_contig_12006          -------------------VVATIFGNVEA----------K---------PGWLKKQLKS
gut_contig_19714             -----------------ILVVATIFGSTEA----------K---------PGWLKKQLKS
abdomen_contig_18046         -------------------VVATIFGNAEA----------K---------PGWLKKQLKS
antennae_contig_44920        ----------------------------------------K---------PGWLKKQLKS
head_contig_12656            ----------------------------------------K---------PGWLKKQLKS
legs_contig_33582            ----------------------------------------K---------PGWLKKQLKS
legs_contig_4480             ----------------------------EA----------K---------PGWLKKQLKS
snout_contig_13632           ----------------------------------------K---------PGWLKKQLKS
snout_contig_15631           ----------------------------------------K---------PGWLKKQLKS
thorax_contig_12294          ----------------------------------------K---------PGWLKKQLKS
wings_contig_24175           ----------------------------------------K---------PGWLKKQLKS
head_contig_1777             -----RRDIMEP-------LLRI---------------RRA---------GGYASASSH-
thorax_contig_875            -----RRDIMEP-------LLRI---------------RRA---------GGYASASSH-
wings_contig_282             -----RRDIMEP-------LLRI---------------RRA---------GGYASASSH-
legs_contig_679              -----RRDIMEP-------LLRI---------------RRA---------GGYASASSH-
gut_contig_1217              -----RRDIMEP-------LLRI---------------RRA---------GGYASASSH-
abdomen_contig_7701          -----RRDIMEP-------LLRI---------------RRA---------GGYASASSH-
antennae_contig_4063         -----RRDIMEP-------LLRI---------------RRA---------GGYASASSH-
gut_contig_631               -----RRDIMEP-------LLRI---------------RRA---------GGYASASSH-
snout_contig_872             -----RRDIMEP-------LLRI---------------RRA---------GGYASASSH-
thorax_contig_5237           -----RRDIMEP-------LLRI---------------RRA---------GGYASASSH-
legs_contig_17294            -----RRDIMEP-------LLRI---------------RRA---------GGYASASSH-
wings_contig_264             -----RRDIMEP-------LLRI---------------RRA---------GGYASASSH-
```

```
wings_contig_247            -----RRDIMEP-------LLRI---------------RRA---------GGYASASSH-
thorax_contig_5238          -----RRDIMEP-------LLRI---------------RRA---------GGYASASSH-
legs_contig_738             -----RRDIMEP-------LLRI---------------RRA---------GGYASASSH-
legs_contig_678             -----RRDIMEP-------LLRI---------------RRA---------GGYASASSH-
legs_contig_236             -----RRDIMEP-------LLRI---------------RRA---------GGYASASSH-
legs_contig_1006            -----RRDIMEP-------LLRI---------------RRA---------GGYASASSH-
head_contig_2691            -----RRDIMEP-------LLRI---------------RRA---------GGYASASSH-
head_contig_1779            -----RRDIMEP-------LLRI---------------RRA---------GGYASASSH-
gut_contig_630              -----RRDIMEP-------LLRI---------------RRA---------GGYASASSH-
antennae_contig_5028        -----RRDIMEP-------LLRI---------------RRA---------GGYASASSH-
antennae_contig_304         -----RRDIMEP-------LLRI---------------RRA---------GGYASASSH-
abdomen_contig_7702         -----RRDIMEP-------LLRI---------------RRA---------GGYASASSH-
gut_contig_1216             -----RRDIMEP-------LLRI---------------RRA---------GGYASASSH-
head_contig_25197           PL--------ARYP-------------QLIRLRVLPSRLI-----------------RGP
snout_contig_3247           PL--------ARYP-------------QLIRLRVLPSRLI-----------------RGP
head_contig_10144           PL--------ARYP-------------QLIRLRVLPSRLI-----------------RGP
abdomen_contig_1463         PL--------ARYP-------------QLIRLRVLPSRLI-----------------RGP
head_contig_4692            PL--------ARYP-------------QLIRLRVLPSRLI-----------------RGP
wings_contig_37004          --------------------FQRILYQPTVLLDSVGQITV-----------------PGV
antennae_contig_20969       DF--------EDLDIYFKGNLTDN-----------KTINY-----------------TKK
fat-body_contig_6280        IF--------TGVQ----------------------------------------------
abdomen_contig_17815        ---------------------------STVLLNIAITNKI-----------------TKK
legs_contig_15521           LF--------TAFSTFFKCFFNQWCFASTVLLNIAITNKI-----------------TKK
wings_contig_9480           ----------------VIHVVKHMMATAAAA-----TTAL----------TPLAIGLVGL
head_contig_21464           ----------------PTSLIAA----------------------------AWFREALNI
legs_contig_59268           ----------------LTELLRSFFGGTDGG-----GSDI----------GAFAGSLVGA
snout_contig_65174          ----------------LTELLRSFFGGTDGG-----GSDI----------GAFAGSLVGA
abdomen_contig_3971         ------------------------LPAPEQ-----------------------LNWQ---
head_contig_8773            -------------------------PAPEQ-----------------------LNWQ---
abdomen_contig_9129         --------------------LACAFPAPKQ-----------------------LNWQ---
head_contig_9541            -----------------------------------ESEEI----------SEEENEN---
snout_contig_6309           -------------------------------------EEI----------SEEENEN---
legs_contig_20987           ----------------------------------------------------EENEN---
legs_contig_6429            -----------------------------------ESEDVS---------NEETEDH---
wings_contig_34073          --------------------------------------------------------E---
head_contig_27719           ------------------MCIL--------------------------------------
wings_contig_21115          LM------FEKTEF-GENACLQAMCEVDSG-----------------------C------
wings_contig_145            -----------------DTCIHCICLHESG-----------------------C------
wings_contig_9037           ---------------ISDDCLGCICHIESG-----------------------C------
wings_contig_12979          ----------ENEG-PSRKCLDCICYASST-----------------------CN-----
wings_contig_24016          ---------AEGQD-STRKCLDCMCYASTK-----------------------CD-----
abdomen_contig_9311         --------------------MRCLCHVATE-----------------------CD-----
antennae_contig_3005        --------------------MRCLCHVATE-----------------------CD-----
head_contig_7167            VFNQDENSILKLPN-LDRQCMRCLCHVATE-----------------------CD-----
legs_contig_12571           VFNQDENSILKLPN-LDRQCMRCLCHVATE-----------------------CD-----
thorax_contig_6557_ORF      VFNQDENSILKLPN-LDRQCMRCLCHVATE-----------------------CD-----
thorax_contig_4396          ----------DDLP-VTQQCLGCICEAISA-----------------------CN-----
wings_contig_254            ----------DDLP-VTQQCLGCICEAISA-----------------------CN-----
wings_contig_13576          --------------------------------HPVDPQTIR---------FPYKIAL---
snout_contig_8907           --------AIEDEE-DIYRQ----------------------------------------
snout_contig_51353          --------MAENTS-LIRVKVDCFLPPSVVPFPPLPSTVLR---------NPVINEPESN
head_contig_92              LL------QHGDVN-VIAVD-WSFYAGADY----ISAVAK---------VPYVGQTVAS
head_contig_1114            ------NGIIQGYK-VTYHSVGEWYDNDDQ-----QTKII-----------NQVRTT---
head_contig_1035            ---TSQVTDLGDFT-LINEG------EEEI-----PMAIP-----------DMVGEE---
fat-body_contig_22298       -----------------------------------RSLPP-----------AEVVSG---
gut_contig_24519            --------------------------AALT-----QAAAI-----------KEVD-----
head_contig_21158           ---------------------------ALT-----QAAAI-----------KEV------
head_contig_25408           -------------------------------------AAI-----------KEV------
head_contig_29881           ------------------------------------------------------EDA---
fat-body_contig_5939        -------------------------------------AAI-----------KEIEDA---
gut_contig_19950            -------------------------------------AAI-----------KEIEDA---
thorax_contig_22215         ------------------------------------SVLL-----------TENE-----
gut_contig_34426            LGRMEKITIMDGFT-LIKSK------SNET-----NSNII-----------TEKD-----
wings_contig_8642           LGRMEKITIMDGFT-LIKSK------SNET-----NSTII-----------TEND-----
wings_contig_20348          LLVL--------------------------------------------------------

thorax_contig_11735_ORF     --P-------------------GIGTLSHKGIILDN-------------------RNHQL
head_contig_13019           -----------------------------EELKEDN-------------------RNHQL
antennae_contig_12759       ------------------------------EELKDN-------------------RNHQL
```

```
legs_contig_16152             ----------------------------------DN-------------------RNHQL
snout_contig_10012            --P-------------------GIGTLSQKGTIVDN-------------------RSHQL
wings_contig_35199            --P-------------------GIGTLSQKGTIVDN-------------------RSHQL
legs_contig_56079             --P-------------------GIGTLSQKGTIVDN-------------------RSHQL
head_contig_20645             --P-------------------GIGTLSQKGTIVDN-------------------RSHQL
head_contig_22785             --P-------------------GIGTLSQKGTIVDN-------------------RSHQL
gut_contig_26424              --P-------------------GIATISHTGTILNN-------------------PNHYI
antennae_contig_36996         -------------------------TISHTGTILNN-------------------PNHYI
snout_contig_1435             --P-------------------GIATISHTGTILNN-------------------PNHYI
thorax_contig_5468            --P-------------------GIATISHTGTILNN-------------------PNHYV
snout_contig_1436             --P-------------------GIATISHTGTILNN-------------------PNHYV
head_contig_18056             --P-------------------GIATISHTGTILNN-------------------PNHYV
antennae_contig_18354         --P-------------------GIATISHTGTILNN-------------------PNHYV
gut_contig_34289              --P-------------------GIATISHTGTILNN-------------------PNHYV
legs_contig_18143             --P-------------------GIATISHTGTILNN-------------------PNHYV
snout_contig_25043            --P-------------------GIATISHTGTILNN-------------------PNHYV
wings_contig_10243            --P-------------------GILTVSHKGTISNN-------------------ENHRL
legs_contig_25911             --P-------------------GILTVSHKGTISNN-------------------ENHRL
abdomen_contig_20364          --P-------------------GILTVSHKGTISNN-------------------ENHRL
legs_contig_11863             --P-------------------GILTVSHKGTISNN-------------------ENHRL
thorax_contig_12619_ORF       --P-------------------GILTVSHKGTISNN-------------------ENHRL
head_contig_3380              --P-------------------GILTVSHKGTILDN-------------------KNHQL
snout_contig_16031            --P-------------------GILTVSHKGTILDN-------------------KNHQL
gut_contig_12195              --P-------------------GILTVSHKGTILDN-------------------NKHQL
wings_contig_17859            --P-------------------GILTVSHKGTILDN-------------------NKHQL
snout_contig_8906             --P-------------------GILTVSHKGTILDN-------------------NKHQL
head_contig_11479             --------------------------VSHKGTILDN-------------------NKHQL
legs_contig_12933             --P-------------------GILTVSHKGTILDN-------------------NKHQL
snout_contig_6533             --P-------------------GILTVSHKGTILDN-------------------NKHQL
head_contig_7710              -------------------------TVSHKGTISNN-------------------ENHRL
head_contig_12473             --P-------------------GILTVSHKGTISNN-------------------ENHRL
head_contig_7109              --P-------------------GILTVSHKGTILDN-------------------KNHQL
fat-body_contig_18846         --P-------------------GILTVSHKGTILDN-------------------KNHQL
legs_contig_1172              --P-------------------GILTVSHKGTILDN-------------------KNHQL
abdomen_contig_18023          -----------------------------ATCQL---------GKFR-------------
fat-body_contig_15296         -----------DFE-TPS--HEGRSRVKRATCQL---------GKFR-------------
head_contig_19818             -----------------------------ATCQL---------GKFR-------------
legs_contig_14899             -----------------------------ATCQL---------GKFR-------------
legs_contig_7206              -----------------------------ATCQL---------GKFR-------------
snout_contig_25846            -----------------------------ATCQL---------GKFR-------------
snout_contig_6410             -----------------------------ATCQL---------GKFR-------------
fat-body_contig_11849         -----------DFE-TPS--HEGRSRVKRATCQL---------GKFR-------------
head_contig_18717             -----------------------------ATCQL---------GKFR-------------
antennae_contig_25540         -----------------------------ATCQL---------GKFR-------------
antennae_contig_54400         -----------------------------ATCQL---------GKFR-------------
fat-body_contig_8241          -----------------------------ATCQL---------GKFR-------------
legs_contig_14898             -----------------------------ATCQL---------GKFR-------------
legs_contig_7207              -----------------------------ATCQL---------GKFR-------------
wings_contig_17449            -----------------------------ATCQL---------GKFR-------------
thorax_contig_3348            -----------------------------ATCDL---------LSFE-------------
legs_contig_22292             -----------PEEELTQ--VSEHSRFKRATCDL---------LSFE-------------
antennae_contig_9625          -----------------------------ATCDL---------LSFE-------------
head_contig_1816              -----------DFQ-DDL--AEGPVRVKRATCDL---------LSFE-------------
legs_contig_12290             -----------------------------ATCDL---------LSFE-------------
snout_contig_94               -----------DFQ-DDL--AEGPVRVKRATCDL---------LSFE-------------
wings_contig_501              -----------------------------ATCDL---------LSFE-------------
gut_contig_33663              -----------------------------ATCDL---------LSFE-------------
legs_contig_8953              -----------------------------ATCDL---------LSFE-------------
snout_contig_14149            -----------------------------ATCDL---------LSFE-------------
snout_contig_4937             -----------FEEELIQ--VSEHGRVKRATCDL---------LSFE-------------
head_contig_7282              ---------------------------------L---------LSFE-------------
snout_contig_809              -----------PEEELTQ--VSEHSRFKRATCDL---------LSFE-------------
wings_contig_1880             -----------------------------ATCDL---------LSFE-------------
wings_contig_3598             -----------------------------ATCDL---------LSFE-------------
fat-body_contig_10700         -----------------------------ATCDL---------LSFE-------------
abdomen_contig_11291          -----------------------------ATCDL---------LSFE-------------
head_contig_7550              -----------------------------ATCDL---------LSFE-------------
thorax_contig_10906           -----------LEEELTQ--VSEHGRVKRATCDL---------LSFE-------------
head_contig_5676              -----------FEEELIQ--VSEHGRVKRATCDL---------LSFE-------------
antennae_contig_42989         -----------------------------ATCDL---------LSFE-------------
```

```
antennae_contig_49987     -----------------------------ATCDL---------LSFE-------------
fat-body_contig_12309     -----------FEEELIQ--VSEHGRFKRATCDL---------LSFE-------------
head_contig_7170          -----------------------------ATCDL---------LSFE-------------
legs_contig_5967          -----------------------------ATCDL---------LSFE-------------
wings_contig_6351         -----------------------------ATCDL---------LSFE-------------
fat-body_contig_9117      VGKKL-------------------------------------------------------
legs_contig_67134         -EKAVKRVRDRAKENLGVVA--GYVGVAGQLGLLPGR-----------------------
wings_contig_11242        LEKAVRRVRDRARENLPLVS--DYVGVAAQAGLIKGG-----------------------
abdomen_contig_11013      AENGVRRVRDRAQENLPVVA--GYVGVAAQAGLLPGK-----------------------
fat-body_contig_4257      AENGVRRVRDRAQENLPVVA--GYVGVAAQAGLLPGK-----------------------
wings_contig_33172        AENGVRRVRDRAQENLPVVA--GYVGVAAQAGLLPGK-----------------------
wings_contig_26325        AENGVRRVRDRAQENLPVVA--GYLGVAAQAGLLPGK-----------------------
thorax_contig_19456       VEKAVRRVRDRAQENLPLVQ--GYVGVAAQAGLLPGR-----------------------
gut_contig_36950          VEKAVRRVRDRAQENLPLVQ--GYVGVAAQAGLLPGR-----------------------
wings_contig_3463         VEKAVRRVRDRAQENLPLVQ--GYVGVAAQAGLLPGR-----------------------
head_contig_10176         VEKSVRRVRDRAQENLPLVA--GYVGVAAQAGLVPGK-----------------------
head_contig_8472          VEKSVRRVRDRAQENLPLVA--GYVGVAAQAGLVPGK-----------------------
wings_contig_19057        VEKSVRRVRDRAQENLPLVA--GYVGVAAQAGLVPG------------------------
wings_contig_20315        VEKSVRRVRDRAQENLPLVA--GYVGVAAQAGLVPGK-----------------------
thorax_contig_12006       VEKGVRRVRDRAQENLPLVA--GYVGVAAQAGLVPGK-----------------------
gut_contig_19714          VEKGVRRVRDRAQENLPLVA--GYVGVAAQAGLVPGK-----------------------
abdomen_contig_18046      VEKGVRRVRDRAQENLPLVA--GYVGVAAQAGLVPGK-----------------------
antennae_contig_44920     VEKGVRRVRDRAQENLPLVA--GYVGVAAQAGLVP-------------------------
head_contig_12656         VEKGVRRVRDRAQENLPLVA--GYVGVAAQAGLV--------------------------
legs_contig_33582         VEKGVRRVRDRAQENLPLVA--GYVGVAAQAGL---------------------------
legs_contig_4480          VEKGVRRVRDRAQENLPLVA--GYVGVAAQAGLVPGK-----------------------
snout_contig_13632        VEKGVRRVRDRAQENLPLVA--GYVGVAAQAGLVPGK-----------------------
snout_contig_15631        VEKGVRRVRDRAQENLPLVA--GYVGVAAQAGLVP-------------------------
thorax_contig_12294       VEKGVRRVRDRA------------------------------------------------
wings_contig_24175        VEKGVRRVR---------------------------------------------------
legs_contig_15450         -----------------AGDWS--SDVCSSDPKI--------------------------
legs_contig_9154          --------------------------------KI--------------------------
antennae_contig_22825     -----------------------------SEAKV--------------------------
wings_contig_11135        -------------------MLT--LAVYECNCKV--------------------------
antennae_contig_8550      -------------------MLT--LAVYECNCKV--------------------------
antennae_contig_4078      --------------------------------KV--------------------------
head_contig_13466         --------------------------------KV--------------------------
head_contig_4570          --------------------------------KV--------------------------
legs_contig_3439          --------------------------------KV--------------------------
legs_contig_3479          -------------------MLT--LAVYECNCKV--------------------------
wings_contig_7149         --------------------------------KV--------------------------
gut_contig_26997          --------------------------------KV--------------------------
abdomen_contig_2669       --------------------------------KV--------------------------
abdomen_contig_2430       --------------------------------KV--------------------------
head_contig_10969         --------------------------------KV--------------------------
gut_contig_9902           --------------------------------KV--------------------------
legs_contig_248           ---------------------------------YVP-------NVAQGL-----------
wings_contig_96           ---------------------------QYYGGDYVP-------NVAQGL-----------
gut_contig_1266           ---------------------------------ASS-------HASASA-----------
wings_contig_310          -------------------------------------------HASASA-----------
head_contig_1777          -----------ASASASASSGSGG-GGQYYGG--AP-------QAFAG------------
thorax_contig_875         -----------ASASASASSGSGG-GGQYYGG----------------------------
wings_contig_282          -----------ASASASASSGSGG-GGQYYGG--AP-------QAFAG------------
legs_contig_679           -----------ASASASASSGSGG-GGQYYGGAGAP-------QAFAG------------
gut_contig_1217           -----------ASASASASSGSGG-GGQYYGGAGAP-------QAFAG------------
abdomen_contig_7701       -----------ASASASASSGSGG-GGQYYGGAGAP-------QAFAG------------
antennae_contig_4063      -----------ASASASASSGSGG-GGQYYGGAGAP-------QAFAG------------
gut_contig_631            -----------ASASASASSGSGG-GGQYYGGAGAP-------QAFAG------------
snout_contig_872          -----------ASASASASSGSGG-GGQYYGGAGAP-------QAFAG------------
thorax_contig_5237        -----------ASASASASSGSGG-GGQYYGGAGAP-------QAFAG------------
legs_contig_17294         -----------ASASASASSGSGGGNGQYYGGAGAP-------QAFAGA-----------
wings_contig_264          -----------ASASASASSGSGGGNGQYYGGDYVP-------NVAQGL-----------
wings_contig_247          -----------ASASASASSGSGGGNGQYYGGDYVP-------NVAQGL-----------
thorax_contig_5238        -----------ASASASASSGSGGGNGQYYGGAGAP-------QAFAGA-----------
legs_contig_738           -----------ASASASASSGSGGGNGQYYGGDYVP-------NV--AQ-----------
legs_contig_678           -----------ASASASASSGSGGGNGQYYGGAPQA-------FA--GA-----------
legs_contig_236           -----------ASASASASSGSGGGNGQYYGGAPQA-------FA--GA-----------
legs_contig_1006          -----------ASASASASSGSGGGNGQYYGGDYVP-------NV--AQ-----------
head_contig_2691          -----------ASASASASSGSGGGNGQYYGGAPQA-------FA--GA-----------
head_contig_1779          -----------ASASASASSGSGGGNGQYYGGAPQA-------FA--GA-----------
```

```
gut_contig_630               -----------ASASASASSGSGGGNGQYYGGDYVP-------NV--AQ-----------
antennae_contig_5028         -----------ASASASASSGSGGGNGQYYGGAPQA-------FA--GA-----------
antennae_contig_304          -----------ASASASASSGSGGGNGQYYGGD---------------------------
abdomen_contig_7702          -----------ASASASASSGSGGGNGQYYGGAPQA-------FA---------------
gut_contig_1216              -----------ASASASASSGSGGGNGQYYGG----------------------------
wings_contig_11007           --------------------------------KVFG------------------------
head_contig_25197            IMKTVAVF--------ALFLLIGLGTVHDTESKVFT------------------------
snout_contig_3247            IMKTVAVF--------AAFVLIGLGNVHETESKVFT------------------------
head_contig_10144            IMKTVAVF--------PAFVLIGMGNVHETESKVFT------------------------
abdomen_contig_1463          IMKTVAVF--------AAFVLIGLGNVHETESKVFT------------------------
wings_contig_1696            --------------------------------KVFT------------------------
fat-body_contig_1889         --------------------------------KVFT------------------------
head_contig_4692             IMKTVAVF--------AAFVLIGLGNVHETESKVFT------------------------
thorax_contig_703_ORF        --------------------------------KVFT------------------------
wings_contig_37004           LCP---------------------------------------------------------
antennae_contig_20969        IIPFSTEF--------SQNDLSVENNLTS-TTQIST------------------------
fat-body_contig_6280         ------------------------------------------------------------
abdomen_contig_17815         IIKILITV--------Q---------LQK-YTTI--------------------------
legs_contig_15521            IIKILITV--------Q---------LQK-YTTI--------------------------
wings_contig_9480            I-----------------------------------------------------------
head_contig_21464            IIRGIT------------GIIWGK----R-------------------------------
wings_contig_67750           ----------------GAGILSGK----IVTGILPALS-----GPPDGDDSMTSTKAPQL
legs_contig_59268            IIKGIAN----PPGAKGAGILSGK----IVTGILPALS-----GPPDGDDSMTSTKAPQL
snout_contig_65174           IIKGIAN----PPGAKGAGILSGK----IVTGILPALS-----GPPDGDDSMTSTKAPQL
legs_contig_31199            ------------------AKPLGPGATCKKDGSL-GTCKSNICVQEKNAD----------
abdomen_contig_3971          -----------PPEQKSEAQLYGEGGFHHRDGVG-VV-----GFVNPNGS----------
head_contig_8773             -----------PPEQKSEAQLYGEGGFHHRDGVG-VV-----GFVNPNGS----------
abdomen_contig_17826         -----------------QAQGFGGGGFQHRDGGG-AA-----GFVNPDGS----------
abdomen_contig_9129          -----------PTEQKSQAQGFGGGGFQHRDGGG-AA-----GFVNPDAS----------
head_contig_9541             -------------------------HE---------------------------------
snout_contig_6309            -------------------------HE---------------------------------
legs_contig_20987            -------------------------HE---------------------------------
legs_contig_6429             -------------------------EV---------------------------------
gut_contig_2593              -------------------------SA---------------------------------
wings_contig_34073           -------------------------IV---------------------------------
head_contig_27719            -------------------------------------C-----AIFHS------------
wings_contig_21115           -----------------------VPVGCSTDVHGRVGC-----GYFNLN-----------
wings_contig_145             -----------------------KAIGCHMDV-GSLSC-----GYYQIK-----------
wings_contig_9037            -----------------------RPVGCHMDV-GSLSC-----GYYQIK-----------
wings_contig_12979           -----------------------EKVGCFNGGPESYFC-----GPYQIS-----------
wings_contig_24016           -----------------------EKIGCHNAGPESYFC-----GPYQIT-----------
abdomen_contig_9311          -----------------------LTKGCDK-----GYC-----GPYKIS-----------
antennae_contig_3005         -----------------------LTKGCDK-----GYC-----GPYKIS-----------
head_contig_7167             -----------------------LTKGCDK-----GYC-----GPYKIS-----------
legs_contig_12571            -----------------------LTKGCDK-----GYC-----GPYKIS-----------
thorax_contig_6557_ORF       -----------------------LTKGCDK-----GYC-----GPYKIS-----------
thorax_contig_4396           -----------------------TSSSCSG-----DVC-----GPFRIT-----------
wings_contig_254             -----------------------TSSSCSG-----DVC-----GPFRIT-----------
wings_contig_13576           --SKNEVA---PGEDIEITISGRSYLGVFL-------------Q-FRNS-----------
snout_contig_51353           -----------W-GALVLVIESSPSGGPLKAGRIPVTI-----FPDKIP-----------
head_contig_92               FIDGIIDS---FGYSLDLVILMGHSLGAHVAGYA-----------GKN------------
head_contig_1114             -ISGLRKF---TNYSITVLAYTGSGDGVRSPSVF---C-----QTEED------------
head_contig_1035             -DIGA------PSEKMPVEKPTEEGLLLDK--------------HEEG------------
fat-body_contig_22298        -PS----------KPPSIR-----------------------------------------
gut_contig_24519             --NEA------AKEPLGRRHH-SLSLSLSLSITY---C-----FSFRS------------
head_contig_21158            -DNEA------PKEPLGVTCN-GHNGGTTVACTV---H-----CMVDG------------
head_contig_25408            -DNEA------PKEPLGVTCN-GHNGGTTVACTV---H-----CMVDG------------
head_contig_29881            -PSSD------DSAPLRITCN-GHDGGTTIPCTV---H-----CMVDG------------
fat-body_contig_5939         -PSSD------DSAPLRITCN-GHDGGTTIPCTV---H-----CMVDG------------
gut_contig_19950             -PSSD------DSAPLRITCN-GHDGGTTIPCTV---H-----CMVDG------------
thorax_contig_22215          -----------IDQNLARSLE-GEGKTLNK--ML---I-------DKA------------
gut_contig_34426             -----------IDENLGRSLD-DKNAVLNK--IL---V-------DKA------------
wings_contig_8642            -----------IDENLGRSLD-DKNAVLNK--IL---V-------DKA------------
wings_contig_20348           -----------------KKYM-GKHNVFPE--MV---L-------QLG------------
wings_contig_9143            -------------------------QTLPK------------------------------


legs_contig_41913            --MGFV---TKDFTRKGLTPDALGGNLGYV----------NKPSGSNLGIGADSIRGYGT
thorax_contig_11735_ORF      SGMGFV---TKDFTRKGLTPDALGGNLGYV----------NKPSGSNLGIGADSIRGYGT
head_contig_13019            SGMGFV---TKDFTRKGLTPDALGGNLRYV----------NKPSGSNLGIGADSIRGYGT
```

```
snout_contig_18124            --MGFV---TKDFTRKGLTPDALGGNLRYV----------NKPSGSNLGIGADSIRGYGT
wings_contig_3386             --MGFV---TKDFTRKGLTPDALGGNLRYV----------NKPSGSNLGIGADSIRGYGT
antennae_contig_12759         SGMGFV---TKDFTRKGLTPDALGGNLRYV----------NKPSGSNLGIGADSIRGYGT
head_contig_18386             --MGFV---TKDFTRKGLTPDALGGNLRYV----------NKPSGSNLGIGADSIRGYGT
antennae_contig_19867         --MGFV---TKDFTRKGLTPDALGGNLRYV----------NKPSGSNLGIGADSKRGYGT
legs_contig_16152             SGMGFV---TKDFTRKGLTPDALGGNLRYV----------NKPSGSNLGIGADSKRGYGT
snout_contig_18125            --MGFV---TKDFTRKGLTPDALGGNLRYV----------NKPSGSNLGIGADSKRGYGT
snout_contig_35068            --MGFV---TKDFTRKGLAPDAFGGSLGYV----------NKPTRSNLGIGADSIRGYGT
snout_contig_10012            NGMGFV---TKDFTRKGLAPDAFGGSLGYV----------NKPTGSNLGIGADSIKGYGT
wings_contig_35199            NGMGFV---TKDFTRKGLAPDAFGGSLGYV----------NKPTGSNLGIGADSIKGYGT
legs_contig_56079             NGMGFV---TKDFTRKGFAPDAFGGSLGYV----------NKPTGSNLGIGADSIRGYGT
head_contig_20645             NGMGFV---TKDFTRKGLAPDAFGGSLGYV----------NKPTGSNLGIGADSIRGYGT
head_contig_22785             NGMGFV---TKDFTRKGLAPDAFGGSLGYV----------NKPTGSNLGIGADSIRGYGT
gut_contig_26424              SGTGYL---SKNLRGGGFAPETYGGNVGYI----------HKPTGSNLGVGADHTRGSGT
antennae_contig_36996         SGTGYL---SKNLRGGGFAPETYGGNVGYI----------HKPTGSNLGVGADHTRGSGT
snout_contig_1435             SGTGYL---SKNLRGGGFAPETYGGNVGYI----------HKPTGSNLGVGADHTRGSGT
thorax_contig_5468            SGTGYL---SKNLRGGGFAPETYGGNVGYI----------HKPTGSNLGVGADHTRGSGT
snout_contig_1436             SGTGYL---SKNLRGGGFAPETYGGNVGYI----------HKPTGSNLGVGADHTRGSGT
head_contig_18056             SGTGYL---SKNLRGGGFAPETYGGNVGYI----------HKPTGSNLGVGADHTRGSGT
antennae_contig_18354         SGTGYL---SKNLRGGGFAPETYGGNVGYI----------HKPTGSNLGVGADHTRGSGT
gut_contig_34289              SGTGYL---SKNLRGGGFAPETYGGNVGYI----------HKPTGSNLGVGADHTRGSGT
legs_contig_18143             SGTGYL---SKNLRGGGFAPETYGGNVGYI----------HKPTGSNLGVGADHTRGSGT
snout_contig_25043            SGTGYL---SKNLRGGGFAPETYGGNVGYI----------HKPTGSNLGVGADHTRGSGT
wings_contig_10243            EGMGYA---QKNLRGHGIEPEVFGGGLKYT----------HIPSESNLKIGADHAPGFGT
legs_contig_25911             EGMGYA---QKNLRGHGIEPEVFGGGLKYT----------HIPSESNLKIGADHAPGFGT
abdomen_contig_20364          EGMGYA---QKNLRGHGIEPEVFGGGLKYT----------HIPSESNLKIGADHAPGFGT
legs_contig_11863             EGMGYA---QKNLRGHGIEPEVFGGGLKYT----------HIPSESNLKIGADHAPGFGT
thorax_contig_12619_ORF       EGMGYA---QKNLRGHGIEPEVFGGGLKYT----------HIPSESNLKIGADHAPGFGT
head_contig_3380              EGMGYA---QKNLRGHGIEPEVFGGGLKYT----------HIPSESNLKIGADHAPGFGT
snout_contig_16031            GGMGYA---QKNLRGHGIEPEVFGGGLKYT----------HIPSESNLKIGADHAPGFGT
gut_contig_12195              DGMGYV---QKNLRGHGIEPEVFGGGLKYT----------HKPSESNLKIGADHARGFGT
antennae_contig_31250         --MGYV---QKNLRGHGIEPEVFGGGLKYT----------HKPSESNLKIGADHARGFGT
wings_contig_26271            --MGYV---QKNLRGHGIEPEVFGGGLKYT----------HKPSESNLKIGADHARGFGT
wings_contig_17859            DGMGYV---QKNLRGHGIEPEVFGGGLKYT----------HKPSESNLKIGADHARGFGT
thorax_contig_8376_ORF        --MGYV---QKNLRGHGIEPEVFGGGLKYT----------HKPSESNLKIGADHARGFGT
snout_contig_8906             DGMGYV---QKNLRGHGIEPEVFG------------------------------------
head_contig_11479             DGMGYV---QKNLRGHGIEPEVFGGGLKYT----------HKPSESNLKIGADHARGFGT
legs_contig_12933             DGMGYV---QKNLRGHGIEPEVFGGGLKYT----------HKPSESNLKIGADHARGFGT
snout_contig_6533             DGMGYV---QKNLRGHGIEPEVFGGGLKYT----------HKPSESNLKIGADHARGFGT
fat-body_contig_23056         --MGYA---QKNLRGHGIEPEVFGGGLKYT----------HIPSNSNLKIGADHAPGLGT
wings_contig_7236             --MGYA---QKNLRGHGIEPEVFGGGLKYT----------HIPSESNLKIGADHAPGFGT
snout_contig_39063            --MGYA---QKNLRGHGIEPEVFGGGLKYT----------HIPSESNLKIGADHAPGFGT
head_contig_7710              EGMGYA---QKNLRGHGIEPEVFGGGLKYT----------HIPSESNLKIGADHAPGFGT
head_contig_15936             --MGYA---QKNLRGHGIEPEVFGGGLKYT----------HIPSESNLKIGADHAPGFGT
antennae_contig_31537         --MGYA---QKNLRGHGIEPEVFGGGLKYT----------HIPSESNLKIGADHAPGFGT
head_contig_12473             EGMGYA---QKNLRGHGIEPEVFGGGLKYT----------HIPSESNLKIGADHAPGFGT
gut_contig_11250              --MGYI---QKNLRGHGIEPEVLGGGLKYT----------HIPSNSNLKIGADHARGFGT
antennae_contig_13155         ------------------------GGLKYT----------HIPSNSNLKIGADHARGLGT
head_contig_7109              GGMGYV---QKNLRGHGIEPEVLGGGLKYT----------HIPSNSNLKIGADHARGLGT
gut_contig_40919              --MGYV---QKNLRGHGIEPEVLGGGLKYT----------HIPSNSNLKIGADHARGLGT
antennae_contig_12922         --MGYV---QKNLRGHGIEPEVLGGGLKYT----------HIPSNSNLKIGADHARGLGT
antennae_contig_17487         ------------------------GGLKYT----------HIPSNSNLKIGADHARGLGT
fat-body_contig_18846         GGMGYV---QKNLRGHGIEPEVLGGGLKYT----------HIPSNSNLKIGADHARGLGT
legs_contig_1172              GGMGYV---QKNLRGHGIEPEVLGGGLKYT----------HIPSNSNLKIGADHARGLGT
thorax_contig_23718           -----------------------GGGLKYT----------HIPSNSNLKIGADHARGLGT
abdomen_contig_18023          ----------------------------------IFGVKYNDSLCAAHCL----------
fat-body_contig_15296         ----------------------------------IFGVKYNDSLCAAHCL----------
head_contig_19818             ----------------------------------IFGVKYNDSLCAAHCL----------
legs_contig_14899             ----------------------------------IFGVKYNDSLCAAHCL----------
legs_contig_7206              ----------------------------------IFGVKYNDSLCAAHCL----------
snout_contig_25846            ----------------------------------IFGVKYNDSLCAAHCL----------
snout_contig_6410             ----------------------------------IFGVKYNDSLCAAHCL----------
abdomen_contig_14676          -------------------------------------MKYNDSLCAAHCL----------
fat-body_contig_11849         ----------------------------------IFGMKYNDSLCAAHCL----------
head_contig_18717             ----------------------------------IFGMKYNDSLCAAHCL----------
antennae_contig_25540         ----------------------------------IFGMKYNDSLCAAHCL----------
antennae_contig_54400         ----------------------------------IFGMKYNDSLCAAHCL----------
fat-body_contig_8241          ----------------------------------IFGMKYNDSLCAAHCL----------
legs_contig_14898             ----------------------------------IFGMKYNDSLCAAHCL----------
legs_contig_7207              ----------------------------------IFGMKYNDSLCAAHCL----------
```

```
wings_contig_17449          ----------------------------------IFGMKYNDSLCAAHCL----------
thorax_contig_3348          ----------------------------------VKGFKLNDSACAAHCL----------
legs_contig_22292           ----------------------------------VKGFKLNDSACAAHCL----------
antennae_contig_9625        ----------------------------------VKGFKLNDSACAAHCL----------
head_contig_1816            ----------------------------------VKGFKLNDSACAAHCL----------
legs_contig_12290           ----------------------------------VKGFKLNDSACAAHCL----------
snout_contig_94             ----------------------------------VKGFKLNDSACAAHCL----------
wings_contig_501            ----------------------------------VKGFKLNDSACAAHCL----------
gut_contig_33663            ----------------------------------AFGIKLNDSACAAHCL----------
legs_contig_8953            ----------------------------------AFGIKLNDSACAAHCL----------
snout_contig_14149          ----------------------------------AFGIKLNDSACAAHCL----------
snout_contig_4937           ----------------------------------AFGIKLNDSACAAHCL----------
head_contig_7282            ----------------------------------AFGIKLNDSACAAHCL----------
snout_contig_809            ----------------------------------AFGIKLNDSACAAHCL----------
wings_contig_1880           ----------------------------------AFGIKLNDSACAAHCL----------
wings_contig_3598           ----------------------------------AFGIKLNDSACAAHCL----------
fat-body_contig_10700       ----------------------------------AFGIKLNDSACATHCL----------
abdomen_contig_11291        ----------------------------------AFGIKLNDSACAAHCL----------
head_contig_7550            ----------------------------------AFGIKLNDSACAAHCL----------
thorax_contig_10906         ----------------------------------AFGIKLNDSACAAHCL----------
head_contig_5676            ----------------------------------AFGIKLNDSACAAHCL----------
antennae_contig_42989       ----------------------------------AFGIKLNDSACAAHCL----------
antennae_contig_49987       ----------------------------------AFGIKLNDSACAAHCL----------
fat-body_contig_12309       ----------------------------------AFGIKLNDSACAAHCL----------
head_contig_7170            ----------------------------------AFGIKLNDSACAAHCL----------
legs_contig_5967            ----------------------------------AFGIKLNDSACAAHCL----------
wings_contig_6351           ----------------------------------AFGIKLNDSACAAHCL----------
legs_contig_15450           ---YSRCEFAKAIKKYGITS--NLGTWTCI---------ASHESNFNTQA----------
legs_contig_21173           -----------------------------------------HESNFNTQA----------
legs_contig_41848           ------------------------------------------------QA----------
legs_contig_9154            ---YSRCEFAKAIKKYGITS--NLGTWTCI---------ASHESNFNTQA----------
wings_contig_30037          --------------------------------------------NFNTQA----------
wings_contig_3613           ------------MKNQGVND--DLGTWTCI---------ASHESNFNTQA----------
snout_contig_11181          --------ICTSNEEPRRND--DLGTWTCI---------ASHESNFNTQA----------
antennae_contig_22825       ---YSPCEFAQAMKNQGVND--DLGTWTCI---------ASHESNFNTQA----------
wings_contig_4225           ------------MKNQGVND--DLGTWTCI---------ASHESNFNTQA----------
legs_contig_3478            ---------------NGIKSIADLGTWTCI---------AFHESRFDTNA----------
head_contig_2467            ------------MKYNGIKSIADLGTWTCI---------AFHESRFDTNA----------
fat-body_contig_17909       -------------------------TWTCI---------AFHESRFDTNA----------
antennae_contig_10005       ----------------------------YI---------TFHESRFDTNA----------
antennae_contig_5138        ------------------------------------------------NA----------
wings_contig_9391           ------------MKYNGIKSIADLGTWTCI---------AFHESRFDTNA----------
wings_contig_11135          ---YGKCEFAKTMKYNGIKSIADLGTWTCI---------AFHESRFDTNA----------
snout_contig_4938           ------------MKYNGIKSIADLGTWTCI---------AFHESRFDTNA----------
legs_contig_3477            ---------------NGIKSIADLGTWTCI---------AFHESRFDTNA----------
legs_contig_32468           -------------------------TWTCI---------AFHESRFDTNA----------
antennae_contig_8550        ---YGKCEFAKTMKYNGIKSIADLGTWTCI---------AFHESRFDTNA----------
antennae_contig_4078        ---YGKCEFAKTMKYNGIKSIADLGTWTCI---------AFHESRFDTNA----------
head_contig_13466           ---YGKCEFAKTMKYNGIKSIADLGTWTCI---------AFHESRFDTNA----------
head_contig_4570            ---YGKCEFAKTMKYNGIKSIADLGTWTCI---------AFHESRFDTNA----------
legs_contig_3439            ---YGKCEFAKTMKYNGIKSIADLGTWTCI---------AFHESRFDTNA----------
legs_contig_3479            ---YGKCEFAKTMKYNGIKSIADLGTWTCI---------AFHESRFDTNA----------
wings_contig_7149           ---YGKCEFAKTMKYNGIKSIADLGTWTCI---------AFHESRFDTNA----------
wings_contig_27298          ------------LKAHGISSRAQIATWTCI---------ASHESGFNTKA----------
gut_contig_26997            ---YNKCAFAKQLKAHGISSRAQIATWTCI---------ASHESGFNTKA----------
abdomen_contig_2669         ---YNKCAFAKQLKAHGISSRAQIATWTCI---------ASHESGFNTKA----------
abdomen_contig_2430         ---YNKCAFAKQLKAHGISSRAQIATWTCI---------ASHESGFNTKA----------
head_contig_10969           ---YTKCEFAQEMKKHGVTAHADLGTWTCI---------ASHESAFNTKA----------
antennae_contig_32201       ------------MKKHGVTSHADLGTWTCI---------ASHESAFNTKA----------
gut_contig_9902             ---YTKCEFAQEMKKHGVTSHADLGTWTCI---------ASHESAFNTKA----------
wings_contig_20241          ---------------------------------GQPSSFAGASSSSNSGV----------
antennae_contig_992         ----------------------------------------GASSSSNAGV----------
legs_contig_248             ---SQIHGQVQDL----------LNNIASGVGAGAPQAFAGASSSSNAGV----------
thorax_contig_2506          -------------------------------GAGAPQAFAGASSSSNAGV----------
thorax_contig_711           ---------------------------------GAPQAFAGASSSSNAGV----------
wings_contig_96             ---SQIHGQVQDL----------LNNIASGVGAGAPQAFAGASSSSNAGV----------
wings_contig_522            ----------------------------VASGVGAPQAFAGASSSSNAGV----------
wings_contig_4999           -----------------------------------PSSFAGASSSSNSGV----------
```

```
legs_contig_1266          ------------------------TAYEISRSDWSSDVCSSDPASSNAGV----------
gut_contig_1266           ---SASSGSGGGN----------GQYYGG-----APQAFAGASSSSNAGV----------
head_contig_914           ---------GGGN----------GQYYGG-----APQAFAGASSSSNAGV----------
wings_contig_310          ---SASSGSGGGN----------GQYYGG-----APQAFAGASSSSNAGV----------
head_contig_1777          -----------------------------------------ASSSSNAGV----------
wings_contig_282          -----------------------------------------ASSSSNAGV----------
legs_contig_679           -----------------------------------------ASSSSNAGV----------
gut_contig_1217           -----------------------------------------ASSSSNAGV----------
abdomen_contig_7701       -----------------------------------------ASSSSNAGV----------
antennae_contig_4063      -----------------------------------------ASSSS--------------
snout_contig_872          -----------------------------------------ASSSSNAGV----------
thorax_contig_5237        -----------------------------------------ASSSSNA------------
legs_contig_17294         ---SSSSNA---------------------------------------------------
wings_contig_264          ---S-----------QIHGQVQ-DLLNNIASGVGAPQAFAGASSSSNAGV----------
wings_contig_247          ---S-----------QIHGQVQ-DLLNNIASGVGAPQAFAGASSS--------------
thorax_contig_5238        ---SSSSNAG--------------------------------------------------
legs_contig_738           ---GLSQ---------IHGQVQ-DLLNNIA------------------------------
legs_contig_678           ---SSSSNAG--------------------------------------------------
legs_contig_236           ---SSSSNAGVGGYGPASGQSY-GGLGSGHDA----------------------------
legs_contig_1006          ---GLSQIHGQ---------V---------------------------------------
head_contig_2691          ---SSSSNAGVGGYGPASGQSY-GGPES--------------------------------
head_contig_1779          ---SSSSNAGVG------------------------------------------------
gut_contig_630            ---GLSQ---------IHGQVQ-DLLNNIARPVLSSV-----------------------
antennae_contig_5028      ---SSSSNAGVGGYGPASGQSY-GG-----------------------------------
wings_contig_11007        -----RCEFVK-LARQN-FPANEINDWTCI---------AQHESSYNSKA----------
head_contig_25197         -----KCGPTKELLNNG-F-----------------------------------------
snout_contig_3247         -----KCGLTKELLNNG-FERTYVGHWV--------------------------------
head_contig_10144         -----KCGLTKELLNNG-FER---------------------------------------
abdomen_contig_1463       -----KCGLTKELLNNG-FERTYVGHWVCL---------VESESAKNTSK----------
wings_contig_1696         -----KCGLTKELLNNG-FERTYVGHWVCL---------VESESAKNTSK----------
fat-body_contig_1889      -----KCGLTKELLNNG-FERTYVGHWVCL---------VESESAKNTSK----------
head_contig_4692          -----KCGLTKELLNNG-FERTYVGHWVCL---------VESESAKNTSK----------
thorax_contig_703_ORF     -----KCGLTKELLNNG-FERTYVGHWVCL---------VESESAKNTSK----------
antennae_contig_20969     -----KKERIDPLL----QNRTTLPDETRN---------PRQEDASSEEE----------
fat-body_contig_6280      ----------TVIL----LEKNVV------------------------------------
abdomen_contig_17815      --------VITYIK----LN----------------------------------------
legs_contig_15521         --------VITYIK----LNRNSIL-----------------------------------
wings_contig_9480         --------------------------A------------AQAFIIAKIALVI--------
head_contig_21464         --------FLNCLLA---------------------------------------------
wings_contig_67750        DSGSFITGFLKTVLGSGGNGTTEGGKK------------QSTFTL--IKLVF--------
legs_contig_59268         DSGSFITG----------------------------------------------------
snout_contig_65174        DSGSFITGFLKTVLGSGGNGTTEGG-----------------------------------
legs_contig_31199         -T-----GKCK-------------------------------------------------
abdomen_contig_3971       -G-----GQVKYGHESG-FGQSYG-----A----------------DVRV----------
head_contig_8773          -G-----GQVKYGHESG-FGQSYG-----A----------------DVRV----------
abdomen_contig_17826      -G-----GQVKYGHQSG-FGQSFG-----A----------------DGRV----------
abdomen_contig_9129       -G-----GQVKYGHQSG-FGQSFG-----A----------------DGRV----------
head_contig_9541          -------------------------EELEP----------RSWSCAVGSV----------
snout_contig_6309         -------------------------EALEP----------RSWSCAVGSV----------
legs_contig_20987         -------------------------EALEP----------RSWSCAVGSV----------
legs_contig_17110         ----------------------------------------------MGTA----------
legs_contig_6429          -------------------------DSIEP----------RSWSCAMGTA----------
gut_contig_2593           -------------------------CFLDY----------NI------------------
wings_contig_28652        -----------------------------------------A------------------
wings_contig_34073        -------------------------NSVAP----------QG------------------
wings_contig_20868        -----------------------------------------S------------------
wings_contig_18351        -----------------------------------------G------------------
wings_contig_35590        -----------------------------------------G------------------
wings_contig_55965        -----------------------------------------G------------------
wings_contig_12722        -----------------------------------------G------------------
wings_contig_9173         -----------------------------------------G------------------
head_contig_27719         ----------------------------LCR--TSYNKPSGNGQCRRRRI----------
wings_contig_21115        ---------ILQFRNCH-HPGKVTGEDESV---AWIRCAEDFACSSECI----------
wings_contig_145          ---------LPYYEDCG-QPGKKSGESTET---AWKRCSDDYSCSTTCV----------
wings_contig_9037         ---------LPYYIDCG-EPGKQPGQSTEA---AWKACADNLSCASTCV----------
wings_contig_12979        ---------RAYWIDAG-QPGN-------V---DHFEKCLNDMSCAERTV----------
wings_contig_24016        ---------HAYWIDAG-KPGN-------V---DHFEKCLKDKHCSETAV----------
abdomen_contig_9311       ---------KLYWKDAK-EVTLPDDDKERA---GAYEDCSISYQCAQKIV----------
antennae_contig_3005      ---------KLYWKDAK-EVTLPDDDKERA---GAYEDCSISYQCAQKIV----------
head_contig_7167          ---------KLYWKDAK-EVTLPDDDKERA---GAYEDCSISYQCAQKIV----------
```

```
legs_contig_12571           ---------KLYWKDAK-EVTLPDDDKERA---GAYEDCSISYQCAQKIV----------
thorax_contig_6557_ORF      ---------KLYWKDAK-EVTLPDDDKERA---GAYEDCSISYQCAQKIV----------
thorax_contig_4396          ---------WAYWADSG-KPTINGESPDAP---QAYAHCAGDTYCAALSV----------
wings_contig_254            ---------WAYWADSG-KPTINGESPDAP---QAYAHCAGDTYCAALSV----------
wings_contig_13576          ------------------ENQAIGSF----------------QIS---------------
snout_contig_8907           -----------------------------------------------DSI----------
snout_contig_51353          ------------------APLAPGGFAIPLI--MAP-----TKLPAKAPI----------
head_contig_92              ------------------LDGNIG-VIIGL----DPAGPLYLEITPASRL----------
head_contig_511             -----------------------------MR------KLRWVLVPV--------------
thorax_contig_4262          -----------------------------MR------KLRWVLVPV--------------
wings_contig_95             -----------------------------MR------KLRWVLVPV--------------
legs_contig_618             -----------------------------MR------KLRWVLVPV--------------
thorax_contig_140           -----------------------------MR------KLRWVLVPV--------------
antennae_contig_2181        -----------------------------MR------KLRWVLVPV--------------
thorax_contig_139           -----------------------------MR------KLRWVLVPV--------------
head_contig_1114            ------------------VPSAPS-DIKAVL--SAPNKILVSWLPPKFS-----------
head_contig_1035            ------------------V------D------------VIYDKAPPEDAVKR--------
gut_contig_24519            ------------------F------FWIGMR--SASADVTVTTIPRRSSL----------
head_contig_21158           ------------------Y------HY-GVC---------------VDGV----------
head_contig_25408           ------------------Y------HY-GVC---------------VDGV----------
head_contig_29881           ------------------Y------HY-GVC---------------KNGI----------
fat-body_contig_5939        ------------------Y------HY-GVC---------------KNGI----------
gut_contig_19950            ------------------Y------HY-GVC---------------KNGI----------
thorax_contig_22215         ------------------S------DIFGAR--S-----IEISIPKIASL----------
gut_contig_34426            ------------------T------SILDSR--T-----MQIFIPKASKE----------
wings_contig_8642           ------------------T------SILDSR--T-----MQIFIPKASKE----------
wings_contig_20348          ------------------Q---------------------------HTTL----------

legs_contig_41913           DLSASGRLNLLQL--PNGNFGVSGHYGRH--------FGH--------RPPPRHN-----
thorax_contig_11735_ORF     DLSASGRLNLLQL--PNGNFGVSGHYGRH--------FGH--------RPPPRHN-----
head_contig_13019           DLSASGRLNLLQL--PNGNFGVSGHYGRH--------FGD--------RPPPRHN-----
snout_contig_18124          DLSASGRLNLLQL--PNGNFGVSGHYGRH--------FGD--------RPPPRHN-----
wings_contig_3386           DLSASGRLNLLQL--PNGNFGVSGHYGRH--------FGD--------RPPPRHN-----
antennae_contig_12759       DLSASGRLNLLQL--PNGNFGVSGHYGRH--------FGH--------RPPPRHN-----
head_contig_18386           DLSASGRLNLLQL--PNGNFGVSGHYGRH--------FGH--------RPPPRHN-----
antennae_contig_19867       DLSASGRLNLLQL--PNGNFGVSGHYGRH--------FGH--------RPPPRHN-----
legs_contig_16152           DLSASGRLNLLQL--PNGNFGVSGHYGRH--------FGH--------RPPPRHN-----
snout_contig_18125          DLSASGRLNLLQL--PNGNFGVSGHYGRH--------FGH--------RPPPRHN-----
snout_contig_35068          DLSASGRVNLFQL--PNGNFGISGHYGRH--------FGH--------RLPTR-P-----
snout_contig_10012          DLSANGRVNLFQL--PNGNFGISGHYGRH--------FGH--------RLPTR-P-----
wings_contig_35199          DLSANGRVNLFQL--PNGNFGISGHYGRH--------FGH--------RLPTR-P-----
legs_contig_56079           DLSANGRVNLFQL--PNGNFGISGHYGRH--------FGH--------RLPTR-P-----
head_contig_20645           DLSANGRVNLFQL--PNGNFGISGHYGRH--------FGH--------RLPTR-P-----
head_contig_22785           DLSANGRVNLFQL--PNGNFGISGHYGRH--------FGH--------RLPTR-P-----
gut_contig_26424            NVHASGTYNFLKT--KNGALGLTGEYG---------------------------------
antennae_contig_36996       NVHASGTYNFLKT--KNGALGLTGEYGRN--------LGG--------P-GG--K-----
snout_contig_1435           NVHASGTYNFLKT--KNGALGLTGEYGRN--------LGG--------P-GG--K-----
thorax_contig_5468          NVHASGTYNFLKT--KNGALGLTGEYGRN--------LGG--------P-GG--K-----
snout_contig_1436           NVHASGTYNFLKT--KNGALGLTGEYGRN--------LGG--------P-GG--K-----
head_contig_18056           NVHASGTYNFLKT--KNGALGLTGEYGRN--------LGG--------P-GG--K-----
antennae_contig_18354       NVHASGTYNFLKT--KNGALGLTGEYGRN--------LGG--------P-GG--K-----
gut_contig_34289            NVHASGTYNFLKT--KNGALGLTGEYGRN--------LGG--------P-GG--K-----
legs_contig_18143           NVHASGTYNFLKT--KNGALGLTGEYGRN--------LGG--------P-GG--K-----
snout_contig_25043          NVHASGTYNFLKT--KNGALGLTGEYGRN--------LGG--------P-GG--K-----
wings_contig_10243          NLKASG------------------------------------------------------
legs_contig_25911           NL----------------------------------------------------------
abdomen_contig_20364        NLKASGTYNMYHS--KNA------------------------------------------
legs_contig_11863           NLKASGTYNMYHS--KNADVGLTGQY----------------------------------
thorax_contig_12619_ORF     NLKASGTYNMYHS--KNADVGLTGQYSRV--------IDT--------P-YGNLK-----
head_contig_3380            NLKASGTY----------------------------------------------------
snout_contig_16031          NLKAS-------------------------------------------------------
gut_contig_12195            KLHADATYNIYHS--KNADVGLTGQYSRV--------IDT--------P-IGNLK-----
antennae_contig_31250       KLHADATYNIYHS--KNADVGLTGQYSRV--------IDT--------P-IGNLK-----
wings_contig_26271          KLHADATYNIYHS--KNADVGLTGQYSRV--------IDT--------P-IGNLK-----
wings_contig_17859          KLHADATYNIYHS--KNADVGLTGQYSRV--------IDT--------P-IGNLK-----
thorax_contig_8376_ORF      KLHADATYNIYHS--KNADVGLTGQYSRV--------IDT--------P-IGNLK-----
head_contig_11479           KLHADATYNIYHS--KNADVGLTGQYSRV--------IDT--------P-IGNLK-----
legs_contig_12933           KLHADATYNIYHS--KNADVGLTGQYSRV--------IDT--------P-IGNLK-----
snout_contig_6533           KLHADATYNIYHS--KNADVGLTGQYSRV--------IDT--------P-IGNLK-----
```

```
fat-body_contig_23056      NLKASGTYNMYHS--KNADLGLTGQYSRV--------IDT--------P-YGNLK-----
wings_contig_7236          NLKASGTYNMYHS--KNADVGLTGQYSRV--------IDT--------P-YGNLK-----
snout_contig_39063         NLKASGTYNMYHS--KNADVGLTGQYSRV--------IDT--------P-YGNLK-----
head_contig_7710           NLKASGTYNMYHS--KNADVGLTGQYSRV--------IDT--------P-YGNLK-----
head_contig_15936          NLKASGTYNMYHS--KNADVGLTGQYSRV--------IDT--------P-YGNLK-----
antennae_contig_31537      NLKASGTYNMYHS--KNADVGLTGQYSRV--------IDT--------P-YGNLK-----
head_contig_12473          NLKASGTYNMYHS--KNADVGLTGQYSRV--------IDT--------P-YGNLK-----
gut_contig_11250           NLKASGTYNIYHS--KNADLGLTGQYSRV--------IDT--------P-IW--------
antennae_contig_13155      NLKASGTYNIYHS--KNADLGLTGQYNRV--------INT--------P-YGNLK-----
head_contig_7109           NLKASGTYNIYHS--KNADLGLTGQYNRV--------INT--------P-YGNLK-----
gut_contig_40919           NLKASGTYNIYHS--KNADLGLTGQYNRV--------INT--------P-YGNLK-----
antennae_contig_12922      NLKASGTYNIYHS--KNADLGLTGQYNRV--------INT--------P-YGNLK-----
antennae_contig_17487      NLKASGTYNIYHS--KNADLGLTGQYNRV--------INT--------P-YGNLK-----
fat-body_contig_18846      NLKASGTYNIYHS--KNADLGLTGQYNRV--------INT--------P-YGNLK-----
legs_contig_1172           NLKASGTYNIYHS--KNADLGLTGQYNRV--------INT--------P-YGNLK-----
thorax_contig_23718        NLKASGTYNIYHS--KNADLGLTGQYNRV--------INT--------P-YGNLK-----
abdomen_contig_18023       ------VIGKKG------------------------------------------------
fat-body_contig_15296      ------VIGKKGGYCNRRNTCICRK-----------------------------------
head_contig_19818          ------VIGKKGGYCNRRNTCICRK-----------------------------------
legs_contig_14899          ------VIGKKGGYCNRRNT----------------------------------------
snout_contig_25846         ------VIGKKGGYCNRRNT----------------------------------------
snout_contig_6410          ------VIGKKGGYCNRRNTCICRK-----------------------------------
abdomen_contig_14676       ------VIGKKGGYCNRRNTCICRK-----------------------------------
fat-body_contig_11849      ------VIGKKGGYCNRRNTCICRK-----------------------------------
head_contig_18717          ------VIGKKGGYCNRRNTCICRK-----------------------------------
antennae_contig_25540      ------VIGKKGGYCNRRNTCIC-------------------------------------
antennae_contig_54400      ------VIGKKGGYCNRRNTCICR------------------------------------
fat-body_contig_8241       ------VIGKKGGYCNRRNT----------------------------------------
legs_contig_14898          ------VIGKKGGYCNRRNT----------------------------------------
wings_contig_17449         ------VIGKKGGYCNRRN-----------------------------------------
thorax_contig_3348         ------TLGKKGGHCNSSKVCVCRNYLQI--------YVLMLFC---------CYFSCLE
legs_contig_22292          ------TLGKKGGHCNSSKVCVCRN-----------------------------------
antennae_contig_9625       ------TLGKKG------------------------------------------------
head_contig_1816           ------TLGKKGGHCNSSKVCVCRN-----------------------------------
legs_contig_12290          ------TLGKKGGHCNSSKVCV--------------------------------------
snout_contig_94            ------TLGKKGGHCNSSKVCVCRN-----------------------------------
wings_contig_501           ------TLGKKGGHCNSSKVCVCRN-----------------------------------
gut_contig_33663           ------KLGRRGGHCNSRKVCIC-------------------------------------
legs_contig_8953           ------KLGRRGGHCNSRKVC---------------------------------------
snout_contig_14149         ------KLGRRGGHCNSRKVCICRR-----------------------------------
snout_contig_4937          ------KLGRRGGHCNSRKVCICRR-----------------------------------
head_contig_7282           ------KLGRRGGHCNSRKVCICRK-----------------------------------
snout_contig_809           ------KLGRRGGHCNSRKVCICRK-----------------------------------
wings_contig_1880          ------KLGRRGGHCNSRKVCICRK-----------------------------------
wings_contig_3598          ------KLGRRGGHCNSRKVCICRK-----------------------------------
fat-body_contig_10700      ------QLGRRGGHCNEEKVCVCRR-----------------------------------
abdomen_contig_11291       ------KLGRRGGHCNDEKVCVC-------------------------------------
head_contig_7550           ------KLGRRGGHCNDEKVCVCRR-----------------------------------
thorax_contig_10906        ------QLGRRGGHCNDEKVCVCRK-----------------------------------
head_contig_5676           ------QLGRRGGHCNDEKVCVCRR-----------------------------------
antennae_contig_42989      ------QLGRRGGHCNDEKVCVCR------------------------------------
antennae_contig_49987      ------QLGRRGGHCNDEKVCVC-------------------------------------
fat-body_contig_12309      ------QLGRRGGHCNDEKVCVCRR-----------------------------------
head_contig_7170           ------QLGRRGGHCNDEKVCVCRR-----------------------------------
legs_contig_5967           ------QLGRRGGHCNDEKVCV--------------------------------------
wings_contig_6351          ------QLGRRGGHCNDEKVC---------------------------------------
legs_contig_15450          ------------------INHQTGDYGIL---Q----ISHLYWCSNSNSPGKACGITCQS
legs_contig_21173          ------------------INHQTGDYGIL---Q----ISHLYWCSNSNSPGKACGITCQS
legs_contig_41848          ------------------INHQTGDYGIL---Q----ISHLYWCSNSNSPGKACGITCQS
legs_contig_9154           ------------------INHQTGDYGIL---Q----ISHLYWCSNSNSPGKACGITCQS
wings_contig_30037         ------------------INHQTGDYGIL---Q----ISHLYWCSNSNSPGKACGITCQS
wings_contig_61704         --------------------HQTGDYGIL---Q----ISHLYWCSNSNSPGKACGITCQS
wings_contig_4577          -----------------------GDYGIF---Q----ISHLFWCFDSDTPGKECGITCQS
wings_contig_3613          ------------------INYDTGDYGIL---Q----ISHLYWCSDSDTPGKECGITCQS
snout_contig_11181         ------------------INYDTGDYGIL---Q----ISHLYWCSDSDTPGKECGITCQS
antennae_contig_22825      ------------------INYDTGDYGIL---Q----ISHLYWCSDSDTPGKECGITCQS
head_contig_24921          --------------------------------Q----ISHLYWCSDSDTPGKECGITCQS
head_contig_26317          ---------------------------------------------------KECGITCQS
legs_contig_10639          ------------------------DYGIL---Q----ISHLYWCSDSDTPGKECGITCQS
wings_contig_4225          ------------------INYDTGDYGIL---Q----ISHLYWCSDSDTPGKECGITCQS
```

```
legs_contig_3478          ------------------INHVTGDYGIL---Q----ISHYFWCSDSNVPGKGCHITCQS
head_contig_2467          ------------------INHVTGDYGIL---Q----ISHYFWCSDSNVPGKGCHITCQS
fat-body_contig_17909     ------------------INHVTGDYGIL---Q----ISHYFWCSDSNVPGKGCHITCQS
antennae_contig_10005     ------------------INHVTGDYGIL---Q----ISHYFWCSDSNVPGKGCHITCQS
antennae_contig_5138      ------------------INHVTGDYGIL---Q----ISHYFWCSDSNVPGKGCHITCQS
legs_contig_6570          -------------------------------------------------PGKGCHITCQS
wings_contig_9391         ------------------INHVTGDYGIL---Q----ISHYFWCSDSNVPGKGCHITCQS
wings_contig_11135        ------------------INHVTGDYGIL---Q----ISHYFWCSDSNVPGKGCHITCQS
snout_contig_4938         ------------------INHVTGDYGIL---Q----ISHYFWCSDSNVPGKGCHITCQS
legs_contig_3477          ------------------INHVTGDYGIL---Q----ISHYFWCSDSNVPGKGCHITCQS
legs_contig_32468         ------------------INHVTGDYGIL---Q----ISHYFWCSDSNVPGKGCHITCQS
antennae_contig_8550      ------------------INHVTGDYGIL---Q----ISHYFWCSDSNVPGKGCHITCQS
antennae_contig_4078      ------------------INHVTGDYGIL---Q----ISHYFWCSDSNVPGKGCHITCQS
head_contig_13466         ------------------INHVTGDYGIL---Q----ISHYFWCSDSNVPGKGCHITCQS
head_contig_4570          ------------------INHVTGDYGIL---Q----ISHYFWCSDSNVPGKGCHITCQS
legs_contig_3439          ------------------INHVTGDYGIL---Q----ISHYFWCSDSNVPGKGCHITCQS
legs_contig_3479          ------------------INHVTGDYGIL---Q----ISHYFWCSDSNVPGKGCHITCQS
wings_contig_7149         ------------------INHVTGDYGIL---Q----ISHYFWCSDSNVPGKGCHITCQS
wings_contig_27298        ------------------INKVTGDYGIL---Q----ISHLYWCSNTNKKGKGCNITCAS
gut_contig_26997          ------------------INKVTGDYGIL---Q----ISHLYWCSNT-------------
abdomen_contig_2669       ------------------INKVTGDYGIL---Q----ISHLYWCSNTNKKGKGCNITCAS
abdomen_contig_21772      ------------------------DYGIL---Q----ISHLYWCSNTNKKGKGCNITCAS
abdomen_contig_2430       ------------------INKVTGDYGIL---Q----ISHLYWCSNTNKKGKGCNITCAS
head_contig_10969         ------------------VNSVSGDYGIL---Q----INHYYWCSTTSTPGKGCNITCAS
antennae_contig_32201     ------------------VNSVSGDYGIL---Q----INHYYWCSTTSTPGKGCNITCAS
gut_contig_9902           ------------------VNSVSGDYGIL---Q----INHYYWCSTTSTPGKGCNITCAS
wings_contig_20241        ---------------GGYGPASGQSYGGLGSGHDAGYNGPILFSRFG-EAEGSGI-----
antennae_contig_992       ---------------GGYGPASGQSYGGLGSGHDAGYNGPILFSRFG-EAEGSGI-----
legs_contig_248           ---------------GGYGPASGQSYGGLGSGHDAGYNGPILFSRFG-EAEGSGI-----
thorax_contig_2506        ---------------GGYGPASGQSYGGLGSGHDAGYNGPILFSRFG-EAEGSGI-----
thorax_contig_711         ---------------GGYGPASGQSYGGLGSGHDAGYNGPILFSRFG-EAEGSGI-----
wings_contig_96           ---------------GGYGPASGQSYGGLGSGHDAGYNGPILFSRFG-EAEGSGI-----
wings_contig_522          ---------------GGYGPASGQSYGGLGSGHDAGYNGPILFSRFG-EAEGSGI-----
wings_contig_4999         ---------------GGYGPASGQSYGGLGSGHDAGYNGPILFSRFG-EAEGSGI-----
legs_contig_760           ---------------GGYGPASGQSYGGLGSGHDAGYNGPILFSRFG-EAEGSGI-----
legs_contig_1266          ---------------GGYGPASGQSYGGLGSGHDAGYNGPILFSRFG-EAEGSGI-----
fat-body_contig_3460      ---------------GGYGPASGQSYGGLGSGHDAGYNGPILFSRFG-EAEGSGI-----
gut_contig_1266           ---------------GGYGPASGQSYGGLGSGHDAGYNGPILFSRFG-EAEGSGI-----
head_contig_914           ---------------GGYGPASGQSYGGLGSGHDAGYNGPILFSRFG-EAEGSGI-----
wings_contig_310          ---------------GGYGPASGQSYGGLGSGHDAGYNGPILFSRFG-EAEGSGI-----
head_contig_1777          ---------------GGYGPASGQSYGGL-------------------------------
wings_contig_282          ---------------GGYGPASGQSYGGLGSGHDAGYNGPI-------------------
legs_contig_679           ---------------GGYG-----------------------------------------
gut_contig_1217           ---------------GGYGPASGQSY----------------------------------
abdomen_contig_7701       ---------------GGYGPASGQSYGGLGSGHDAGYNGPILFSRFG-EAEGSGI-----
snout_contig_872          ---------------GGYGPASGQSYG---------------------------------
wings_contig_264          ---------------GGYGPASGQSYGGLGSGHDAGYNGPILFSRFG-------------
wings_contig_11007        ------------TNRNTNGSTDYGIFQIN----------NYWWCTPG-KYN-GCKVKCSD
abdomen_contig_1463       ------------VTDKADRSKSLGLFQIN----------SKEWCKFS-TAGGKCSMKCED
wings_contig_1696         ------------VTDKADRSKSLGLFQIN----------SKEWCKFS-TAGGKCSMKCED
fat-body_contig_1889      ------------VTEKADRSKSLGLFQIN----------SKEWCKFS-TAGGKCSMKCED
head_contig_4692          ------------VTEKADRSKSLGLFQIN----------SKEWCKFS-TAGGKCSMKCED
thorax_contig_703_ORF     ------------VTEKADRSKSLGLFQIN----------SKEWCKFS-TAGGKCSMKCED
antennae_contig_20969     ------------SSEEEDGN----------------------------------------
wings_contig_9480         --AIIIAVRKFLD---------------S---QK--------------------K----K
wings_contig_67750        --SAITGV---FS---------------S-------------------------------
abdomen_contig_3971       --PIYTNKNRYGE--STVNLGAGADYYRG---VG---GSG-----FG------DK----R
head_contig_8773          --PIYTNKNRYGE--STVNLGAGADYYRG---VG---GSG-----FG------DK----R
abdomen_contig_17826      --PIYSNKNRYGE--STVNLGAGADYHKG--------GGG-----FG------DK----G
abdomen_contig_9129       --PIYSNKNSYGE--STVNLGAGADYHKG--------GGG-----FG------DK----R
head_contig_9541          ----------SF-KGFKSGNLFCEFHCRR---QRHR-GGY-------------CD----R
snout_contig_6309         ----------SF-KGFKSGNLFCEFHCRR---QRHR-GGY-------------CD----R
legs_contig_20987         ----------SF-KGFKSGNLFCEFHCRR---HKHR-GGY-------------CE----R
legs_contig_17110         ----------SF-KGFKSGNLFCEFHCRR---HKHR-GGY-------------CE----R
legs_contig_6429          ----------SF-KGFKSGNLFCEFHCRR---HKHR-GGY-------------CE----R
gut_contig_2593           -------------KGFNLGSSLCSLECRM---LKRN-GGQ-------------CG----L
wings_contig_28652        -------------FVCPMNQALCNTHCRQ---IGRK-GGN-------------CG----G
wings_contig_34073        -------------FGCPFDKYQCHSHCKD---LGRK-GGY-------------CN----W
wings_contig_20868        -------------NGCPFNAHECNQFCVS---RRNK-GGY-------------CG----G
wings_contig_18351        -------------FGCPTNRYECHSHCLS---IGRR-GGY-------------CA----G
```

```
wings_contig_35590             -------------FGCPTNRYQCHSHCLS---IGRR-GGY-------------CA----G
wings_contig_55965             -------------FGCPFNRYECNNHCRS---IKRQ-GGY-------------CA----G
wings_contig_12722             -------------FGCPLNRYQCDSHCKS---LGRK-GGY-------------CA----G
wings_contig_9173              -------------FGCPLNRYQCDSHCKS---LGRK-GGY-------------CA----G
wings_contig_10893             ----------------------------------EERSRPLQWL-FQ------AT----Q
head_contig_27719              ----GQYFIKY-------------------------------------------------
wings_contig_21115             ----RRIASRFRTKC--FGKSDCETMARL---HD---GGA-----NG------CR----T
wings_contig_145               ----QNYYNRYKGLCSSYGFGTCEQMSRL---HN---GGP-----SG------CK----Y
wings_contig_9037              ----RNYMNRYAARCHP--NPTCEVFSRV---HN---GGP-----NG------CK----Y
wings_contig_12979             ----DGYMRKWGQDCDRDNTLTCYDFALM---HK---AGR-----FG------CS----A
wings_contig_24016             ----IQYMNKYGTDCDNDGKVTCDDYARM---HK---AGR-----FG------CS----A
abdomen_contig_9311            ----LNYIAKYGRDCNGDGV----------------------------------------
antennae_contig_3005           ----LNYIAKYGRDCNGDGVTDCDDYM---------------------------------
head_contig_7167               ----LNYIAKYGRDCNGDGVTDCDDYMMI---NF---NGG-----YQ------CH----P
legs_contig_12571              ----LNYIAKYGRDCNGDGVTDCDDYMMI---NF---NGG-----YQ------CH----P
thorax_contig_6557_ORF         ----LNYIAKYGRDCNGDGVTDCDDYMMI---NF---NGG-----YQ------CH----P
thorax_contig_4396             ----QGYMNKFQQDCNGDGKIDCD------------------------------------
wings_contig_254               ----QGYMNKFQQDCNGDGKIDCDDFASI---HK---IGG-----YG------CK----G
wings_contig_13576             ------PDDKYFKTVKCHENKLGAATHKN---KEDKKDKVIVW-----------------
snout_contig_8907              -----------------VEDEAGQQYYLV---PL---SRSKR------------------
snout_contig_51353             ----S------------DPP----------------------------------------
head_contig_92                 ----NATDAKYVQAIHTNAMVLGVNYKLA-------------------------------
head_contig_511                -----Q--------------KRGFRFLRL---HR---IWRIGWVRCI------LH----R
thorax_contig_4262             -----Q--------------KHGFRFLRL---HR---IWRIGWVRCI------LH----R
wings_contig_95                -----Q--------------KHGFRFLRL---HR---IWRIGWVRCI------LH----R
legs_contig_618                -----Q--------------KHGFRFLRL---HR---IWRIGWVRCI------LH----R
thorax_contig_140              -----Q--------------KHGFRFLRL---HR---IWRIGWVRCI------LH----R
antennae_contig_2181           -----Q--------------KRGFRFLRL---HR---IWRIGWVRCI------LH----R
thorax_contig_139              -----Q--------------KRGFRFLRL---HR---IWRIGWVRCI------LH----R
head_contig_1114               -----------------NGPLTGYTFYMS---VM---EDGQEEG--T------HK----R
head_contig_1035               --VLTEDDGKI---IKGHKPS------------K---VFE--------------------
fat-body_contig_22298          -----------------RG---SVTTLRN---TR---AS---------------------
gut_contig_24519               ----CQ---------VRLKAYRYVDIYTA---TS---AYR--------------------
head_contig_21158              ----CH---------CKN------------------------------------------
head_contig_25408              ----CH---------CKN------------------------------------------
head_contig_29881              ----CH---------CKN------------------------------------------
fat-body_contig_5939           ----CH---------CKN------------------------------------------
gut_contig_19950               ----CH---------CK-------------------------------------------
thorax_contig_22215            ----VKTEGRS---EHHHKNDTYYTYYVE---RR---RRR--------------------
gut_contig_34426               ----VS-EGRS---DEHHEH----------------------------------------
wings_contig_8642              ----VS-EGRS---DEHH------------------------------------------
wings_contig_20348             ----VKAT--------KAGGSEIVTFKIH---HR---IVD--------------------
wings_contig_9143              ------VC--------HRSTVRYKTF----------------------------------

legs_contig_41913              ------------------------------------NEWGV---FA-GTD-FWF------
thorax_contig_11735_ORF        ------------------------------------NEWGV---FA-GTD-FWF------
head_contig_13019              ------------------------------------NEWGV---FA-GTD-FWF------
snout_contig_18124             ------------------------------------NE----------------------
wings_contig_3386              ------------------------------------NEWGV---FA-GTD-FWF------
antennae_contig_12759          ------------------------------------NEWGV---FA-GTD-FWF------
head_contig_18386              ------------------------------------NEWGV---FA-GTD-FWF------
antennae_contig_19867          ------------------------------------NEWGV---FA-GTD-FWF------
legs_contig_16152              ------------------------------------NEWGV---FA-GTD-FWF------
snout_contig_18125             ------------------------------------NE----------------------
snout_contig_35068             ------------------------------------SDWGV---YA-GTD-FWF------
snout_contig_10012             ------------------------------------SDWGV---YA-GTD-FWF------
wings_contig_35199             ------------------------------------SDWGV---YA-GTD-FWF------
legs_contig_56079              ------------------------------------SDWGV---YA-G------------
head_contig_20645              ------------------------------------SDWGV---YA-GTD-FWF------
head_contig_22785              ------------------------------------SDWGV---YA-GTD-FWF------
antennae_contig_36996          ------------------------------------PNW---------------------
snout_contig_1435              ------------------------------------PNWGV---FL-G------------
thorax_contig_5468             ------------------------------------PNWGV---FL-G------------
snout_contig_1436              ------------------------------------PNWGV---FL-GG-----------
head_contig_18056              ------------------------------------PNWGV---FL-GGK-WNF------
antennae_contig_18354          ------------------------------------PNWGV---FL--------------
gut_contig_34289               ------------------------------------PNWGV---FL-G------------
legs_contig_18143              ------------------------------------PNWGV---FL-GGK-WNF------
snout_contig_25043             ------------------------------------PNWGV---FL-GGK-WNF------
```

```
thorax_contig_12619_ORF     ------------------------------------PNWGV---FL-GGQ-YRF------
gut_contig_12195            ------------------------------------PNWGR---KL-NKL----------
antennae_contig_31250       ------------------------------------PNWGV---FL-EG-----------
wings_contig_26271          ------------------------------------PNWG--------------------
wings_contig_17859          ------------------------------------PNWGV---FL-G------------
thorax_contig_8376_ORF      ------------------------------------PNWGV---FL-GGQ-YRF------
head_contig_11479           ------------------------------------PNWGV---FL-G------------
legs_contig_12933           ------------------------------------PNWGV---FL-GGQ-YRF------
snout_contig_6533           ------------------------------------PNWGV---FL-GGQ-YRF------
fat-body_contig_23056       ------------------------------------PNWGV---YL-GGQ-Y--------
wings_contig_7236           ------------------------------------PNWGV---FL-GGQ-YRF------
snout_contig_39063          ------------------------------------PNWGV---FL-GG-----------
head_contig_7710            ------------------------------------PNWGV---FL-GGQ-YRF------
head_contig_15936           ------------------------------------PNWGV---FL-GGQ-Y--------
antennae_contig_31537       ------------------------------------PNWG--------------------
head_contig_12473           ------------------------------------PNWGV---YL-GGQ-YRF------
antennae_contig_13155       ------------------------------------PNWGV---YL-GGH----------
head_contig_7109            ------------------------------------PNWG--------------------
antennae_contig_12922       ------------------------------------PNWGV---YL-GGQ-YRF------
antennae_contig_17487       ------------------------------------PNWGV---YL-GGQ-YRF------
fat-body_contig_18846       ------------------------------------PNWGV---YL-GGQ-YRF------
legs_contig_1172            ------------------------------------PNWGV---YL-GGQ-YRF------
thorax_contig_23718         ------------------------------------PNWGV---YL-GGQ-YRF------
thorax_contig_3348          --------------------IKLY------------------------------------
legs_contig_15450           LLGDDI-------SRDVACAKKVYGETQRIS-GNGFTAWTTYN-SCKGDQSWWIRNCNV-
legs_contig_21173           LLGDDI-------SRDVACAKKVYGETQRIS-GNGFTAWTTYN-SCKGDQSWWIRNCNV-
legs_contig_41848           LLGDDI-------SRDVACAKKVYGETQRIS-GNGFTAWTTYN-SCKGDQSWWIRNCNV-
legs_contig_9154            LLGDDI-------SRDVACAKKVYGETQRIS-GNGFTAWTTYN-SCKGDQSWWIRNCNV-
wings_contig_30037          LLGDDI-------SRDVACAKKVYGETQRIS-GNGFTAWTTYN-SCKGDQSWWIRNCNV-
wings_contig_61704          LLGDDI-------SRDVACAKKVYGETQRIS-GNGFTAWTTYN-SCKGDQSWWIRNCNV-
wings_contig_4577           LLGDDI-------TEDIICAKKVFSETVAIS-GNGFTAWTTYS-SCGGDQSSWIKECNL-
wings_contig_3613           LLGDDI-------TEDIICAKKVFSETVAIS-GNGFTAWTTYS-SCGGD----------
snout_contig_11181          LLGDDI-------TEDIICAKKVFSETVAIS-GNGFTAWTTYS-SCGGDQSSWIKECNL-
antennae_contig_22825       LLGDDI-------TEDIICAKKVFSETVAIS-GNGFTAWTTYS-SCGGDQSSWIKECNL-
head_contig_24921           LLGDDI-------TEDIICAKKVFSETVAIS-GNGFTAWTTYS-SCGGDQSSWIKECNL-
head_contig_26317           LLGDDI-------TEDIICAKKVFSETVAIS-GNGFTAWTTYS-SCGGDQSSWIKECNL-
legs_contig_10639           LLGDDI-------TEDIICAKKVFSETVAIS-GNGFTAWTTYS-SCGGDQSSWIKECNL-
wings_contig_4225           LLGDDI-------TEDIICAKKVFSETVAIS-GNGFTAWTTYS-SCGGDQSSWIKECNL-
legs_contig_14718           --------------SDIVCAKKIFLETSRGPKKNGFAAWTTYSKYCTGDQRSWVSGCGL-
legs_contig_3478            LLNDDI-------TSDIVCAKKIFNETKNGPKKNGFAAWTTYSKYCTGDQRSWVSGCGL-
head_contig_2467            LLNDDI-------TSDIVCAKKIFNETKNGPKKNGFAAWTTYSKYCTGDQRSWVSGCGL-
fat-body_contig_17909       LLNDDI-------TSDIVCAKKIFNETKNGPKKNGFAAWTTYSKYCTGDQRSWVSGCGL-
antennae_contig_10005       LLNDDI-------TSDIVCAKKIFNETKNGPKKNGFAAWTTYSKYCTGDQRSWVSGCGL-
antennae_contig_5138        LLNDDI-------TSDIVCAKKIFNETKNGPKKNGFAAWTTYSKYCTGDQRSWVSGCGL-
legs_contig_6570            LLNDDI-------TSDIVCAKKIFNETKNGPKKNGFAAWTTYSKYCTGDQRSWVSGCGL-
snout_contig_17238          -----------------VCAKKIFNETKNGPKKNGFAAWTTYSKYCTGDQRSWVSGCGL-
wings_contig_9391           LLNDDI-------TSDIVCAKKIFNETKNGPKKNGFAAWT--------------------
wings_contig_11135          LLNDDI-------TSDIVCAKKIFNETKNGPKK---------------------------
snout_contig_4938           LLNDDI-------TSDIVCAKKIFNETKNGPKKNGFAAWTTYSKYCTGD-----------
legs_contig_3477            LLNDDI-------TSDIVCAKKIFNETKNGPKKNGFAAWTTYSKYCTGDQRS--------
legs_contig_32468           LLNDDI-------TSDIVCAKKIFNETKNGPKKNGFAAWTTYSK----------------
antennae_contig_8550        LLNDDI-------TSDIVCAKKIF------------------------------------
antennae_contig_4078        LLNDDI-------TSDIVCAKKIFNETKNGPKKNGFAAWTTYSKYC--------------
head_contig_13466           LLNDDI-------TSDIVCAKKIFNETKNGPKKNGFAAWTTYSKYCTGDQRSWVSGCGL-
head_contig_4570            LLNDDI-------TSDIVCAKKIFNETKNGPK----------------------------
legs_contig_3439            LLNDDI-------TSDIV------------------------------------------
legs_contig_3479            LLNDDI-------TSDIVCAKKIFNETKNGPKKNGFAAWTTYSKYCTGDQRSWVSGCGL-
wings_contig_7149           LLNDDI-------TSDIVCAKKIFNETKNGPKKNG-------------------------
wings_contig_27298          LLTD--------------------------------------------------------
abdomen_contig_2669         LLTDNI-------SEDIKCAKHIYSETAKLSKKSGFTAWVTYKKYCTGNQSKWISGCRL-
abdomen_contig_21772        LLTDNI-------SEDIKCAKHIYSETAKLSKKSGFTAWVTYKKYCTGNQ----------
abdomen_contig_2430         LLTDNI-------SEDIKCAKHIYSETAKLSKKSGFTAWVTYKKYCTGNQSKWISECRL-
head_contig_10969           LLTDDI-------TVDIKCALHIFSETAKGSTKNGFTAWVTYKKYCTGDQSSWISGCSL-
antennae_contig_32201       LLTDDI-------TVDIKCALHIFSETAKGSTKNGFTAWVTYKKYCTGDQSSWISGCSL-
gut_contig_9902             LLTDDI-------TVDIKCALHIFSETAKGSTKNGFTAWVTYKKYCTGDQSSWISGCSL-
wings_contig_20241          ------------------------------------HVAG----------------QA--
antennae_contig_992         ------------------------------------HVSG----------------QA--
legs_contig_248             ------------------------------------HVSG----------------QA--
thorax_contig_2506          ------------------------------------HVSG----------------QA--
thorax_contig_711           ------------------------------------HVSG----------------QA--
```

```
wings_contig_96              ------------------------------------HVSG----------------QA--
wings_contig_522             ------------------------------------HVSG----------------QA--
wings_contig_4999            ------------------------------------HVSG----------------QA--
legs_contig_760              ------------------------------------HVSG----------------QA--
legs_contig_1266             ------------------------------------HVSG----------------QA--
fat-body_contig_3460         ------------------------------------HVSG----------------QA--
gut_contig_1266              ------------------------------------HVSG----------------QA--
head_contig_914              ------------------------------------HVSG----------------QA--
wings_contig_310             ------------------------------------HVSG----------------QA--
abdomen_contig_7701          ------------------------------------HVSG----------------QA--
abdomen_contig_1463          LLDEDI-------RDDSVCAKKIQ-------RALGFRAWD---------G--WFRSCYRR
wings_contig_1696            LLDEDI-------RDDSVCAKKIQ-------RALGFRAWD---------G--WFRSCYRR
fat-body_contig_1889         LLDEDI-------RDDSVCAKKIQ-------RALGFRAWD---------G--WFRSCYRR
head_contig_4692             LLDEDI-------RDDSVCAKKIQ-------RALGFRAWD---------G--WFRSCYRR
thorax_contig_703_ORF        LLDEDI-------RDDSVCAKKIQ-------RALGFRAWD---------G--WFRSCYRR
wings_contig_9480            PEHPTTP-HTEW-------------------------HSS-----------GWETAEPSP
wings_contig_67750           -ASSLSS-KSDW------------------------------------------------
abdomen_contig_3971          VGLNFN------YRF---------------------------------------------
head_contig_8773             VGLNFN------YRF---------------------------------------------
abdomen_contig_17826         VGLNFN------HRF---------------------------------------------
abdomen_contig_9129          VGLNFN------H-----------------------------------------------
head_contig_9541             --------G------SCRCS----------------------------------------
snout_contig_6309            --------G------SCRCS----------------------------------------
legs_contig_20987            --------G------TCRCG----------------------------------------
legs_contig_17110            --------G------TCRCG----------------------------------------
legs_contig_6429             --------G------TCRCG----------------------------------------
gut_contig_2593              --------N-----NMCICNN---------------------------------------
wings_contig_28652           --------FLR-------------------------------------------------
wings_contig_34073           --------L------VCTCITK--------------------------------------
wings_contig_20868           --------S---LRSSCLCFKK--------------------------------------
wings_contig_18351           --------GIWIIRTTCTCYN---------------------------------------
wings_contig_35590           --------GIWIIRTTCTCYN---------------------------------------
wings_contig_55965           --------S---LRTTCTCFKN--------------------------------------
wings_contig_12722           --------S---LRTTCTCITR--------------------------------------
wings_contig_9173            --------T---LRTTCTCITR--------------------------------------
wings_contig_10893           AL-------------NPCCFLT--------------ILAQ--------------------
head_contig_27719            ---------------RST----------------------------------------RI
wings_contig_21115           ---SKT--AYYWRKVNALCGED--------------CKKP-----------IFRRH----
wings_contig_145             ---SST--LGYWEAIHKCCGCS--------------------------------------
wings_contig_9037            ---PGT--IGYWNKVAGCCSGK--------------HC----------------------
wings_contig_12979           AWVPNT---EYFKKFQACMEPQ--------------AVLD-----------ARKKQQNAS
wings_contig_24016           EWVDNS---DYWKSFKECMQGG--------------VGK---------------------
head_contig_7167             PLNRSDAGRSWLARYKACNPKL--------------RMSG-----------DWS------
legs_contig_12571            PLNRSDAGRSWLARYKACNPKL--------------------------------------
thorax_contig_6557_ORF       PLNRSDAGRSWLARYKACNPKL--------------R-----------------------
wings_contig_254             VPLPE----PYGERYRQCKAIV--------------G-----------------------
wings_contig_13576           -KAPDTAGKYTL-Y-ATV------------------AENG---------GVFWVAKPSGT
snout_contig_8907            -SVP-----------EKK------------------NTQP---------QTDWKVESPGI
head_contig_511              VLIR-VHRSSVL-RPVRI------------------HLLR---------RWSWKRHQRRP
thorax_contig_4262           VLIR-VHRSSAL-RPVRI------------------HLLR---------RWSWKRHQRRP
wings_contig_95              VLIR-VHRSSVL-RPVRI------------------HLLR---------RWSWKRHQRRP
legs_contig_618              VLIR-VHRSSVL-RPVRI------------------HLLR---------RWSWKRHQRRP
thorax_contig_140            VLIR-VHRSSVL-RPVRI------------------HLLR---------RWSWKRHQRR-
antennae_contig_2181         VLIR-VHRSSVL-RPVRI------------------HLLR---------RWSWKRHRSEE
thorax_contig_139            VLIR-VHRSSAL-RPVRI------------------H-----------------------
head_contig_1114             ALSPFTDMHEVI-RAHDT------------------STLQ-----------FWVTASTRI
head_contig_1035             -HSPDT---------VCC------------------LSLK-----------TW-------
fat-body_contig_22298        ---QEL---------SS----------------------H-----------SCR----RF
thorax_contig_22215          -RRPEH---------SHH------------------THHN-----------KWK----KI
gut_contig_34426             ----------------------------------------------------W-------

wings_contig_20241           -------AGPNAAFSS-----SSSSVDSDGKIKYSVQSGKY----------
antennae_contig_992          -------AGPNAAFSS-----SSSSVDSDGKIKYSVQSGKY----------
legs_contig_248              -------AGPNAAFSS-----SSSSVDSDGKIKYSVQSGKY----------
thorax_contig_2506           -------AGPNAAFSS-----SSSSVDSDGKIKYSVQSGKY----------
thorax_contig_711            -------AGPNAAFSS-----SSSSVDSDGKIKYSVQSGKY----------
wings_contig_96              -------AGPNAAFSS-----SSSSVDSDGKIKYSVQSGKY----------
wings_contig_522             -------QGPNAAFSS-----SSSSVDSDGKIKYSVQSGKY----------
wings_contig_4999            -------QGPNAAFSS-----SSSSVDSDGKIKYSVQSGKY----------
```

```
legs_contig_760              -------QGPNAAFSS-----SSSSVDSDGKIKYSVQSGKY----------
legs_contig_1266             -------QGPNAAFSS-----SSSSVDSDGKIKYSVQSGKY----------
fat-body_contig_3460         -------QGPNAAFSS-----SSSSVDSDGKIKYSVQSGKY----------
gut_contig_1266              -------QGPNAAFSS-----SSSSVDSDGKIKYSVQSGKY----------
head_contig_914              -------QGPNAAFSS-----SSSSVDSDGKIKYSVQSGKY----------
wings_contig_310             -------QGPNAAFSS-----SSSSVDSDGKIKYSVQSGKY----------
abdomen_contig_7701          -------AGLPPPPPP-----------------------------------
abdomen_contig_1463          -----------------NLSIPPC---------------------------
wings_contig_1696            -----------------NLSIPPC---------------------------
fat-body_contig_1889         -----------------NLSIPPC---------------------------
head_contig_4692             -----------------NLSIPPC---------------------------
thorax_contig_703_ORF        -----------------NLSIPPC---------------------------
wings_contig_9480            ----------DRR-IS-EAH------------RLA---YKGYRHLHH----
wings_contig_10893           -----------------A-------------MDLAMLSLNRSEHLTL----
head_contig_27719            IAR------------------------------------------------
wings_contig_12979           PIRGPTKTKPQARFIHNPIHRPPAPQGGDDPVRISS--------IIQPKKK
wings_contig_13576           --IN---VA------------------------------------------
snout_contig_8907            --LT---VSHKGTILDNNKH-------QLDGMGY-IQKNLRGHGI------
head_contig_511              --GE---H-------------------------------------------
thorax_contig_4262           --GE---HRHN---ID-HYH-------H-----------------------
wings_contig_95              --GE---HPHR---TR-C---------------------------------
legs_contig_618              --GE---HRHN---I------------------------------------
antennae_contig_2181         --HT---SELQ---SP-AMI-------S-----------------------
head_contig_1114             --GE---GESTKI-IT-VT--------------------------------
fat-body_contig_22298        --GS---GRAR---SS-AVV-------SLHPE-LHVLQLI-----------
thorax_contig_22215          --GE---AILTAI-TA-ALS-------ALVPVAIALIALIAGAALILSKV-
```

Supplementary File S2. Multiple sequence alignments using coloring methods (Clustal Omega) of the peptide sequences from all parts of the body of male and female *R. ferrugineus* adult, both reared in laboratory and collected in field.
